# From Sea to Shining Sea and the Great Plains to Patagonia: A Review on Current Knowledge of Diabetes Mellitus in Hispanics/Latinos in the US and Latin America

**DOI:** 10.3389/fendo.2017.00298

**Published:** 2017-11-10

**Authors:** M. Larissa Avilés-Santa, Uriyoán Colón-Ramos, Nangel M. Lindberg, Josiemer Mattei, Francisco J. Pasquel, Cynthia M. Pérez

**Affiliations:** ^1^National Heart, Lung, and Blood Institute at the National Institutes of Health, Bethesda, MD, United States; ^2^Department of Global Health, Milken Institute School of Public Health, George Washington University, Washington, DC, United States; ^3^Kaiser Permanente Center for Health Research, Portland, OR, United States; ^4^Department of Nutrition, Harvard TH Chan School of Public Health, Boston, MA, United States; ^5^Department of Medicine, Emory University School of Medicine, Atlanta, GA, United States; ^6^University of Puerto Rico Graduate School of Public Health, San Juan, Puerto Rico

**Keywords:** type 2 diabetes mellitus, gestational diabetes, prevention of diabetes, Latinos, Latin America, epidemiology of type 2 diabetes, Hispanics

## Abstract

The past two decades have witnessed many advances in the prevention, treatment, and control of diabetes mellitus (DM) and its complications. Increased screening has led to a greater recognition of type 2 diabetes mellitus (type 2 DM) and prediabetes; however, Hispanics/Latinos, the largest minority group in the US, have not fully benefited from these advances. The Hispanic/Latino population is highly diverse in ancestries, birth places, cultures, languages, and socioeconomic backgrounds, and it populates most of the Western Hemisphere. In the US, the prevalence of DM varies among Hispanic/Latino heritage groups, being higher among Mexicans, Puerto Ricans, and Dominicans, and lower among South Americans. The risk and prevalence of diabetes among Hispanics/Latinos are significantly higher than in non-Hispanic Whites, and nearly 40% of Hispanics/Latinos with diabetes have not been formally diagnosed. Despite these striking facts, the representation of Hispanics/Latinos in pharmacological and non-pharmacological clinical trials has been suboptimal, while the prevalence of diabetes in these populations continues to rise. This review will focus on the epidemiology, etiology and prevention of type 2 DM in populations of Latin American origin. We will set the stage by defining the terms *Hispanic, Latino*, and *Latin American*, explaining the challenges identifying Hispanics/Latinos in the scientific literature and databases, describing the epidemiology of diabetes—including type 2 DM and gestational diabetes mellitus (GDM)—and cardiovascular risk factors in Hispanics/Latinos in the US and Latin America, and discussing trends, and commonalities and differences across studies and populations, including methodology to ascertain diabetes. We will discuss studies on mechanisms of disease, and research on prevention of type 2 DM in Hispanics/Latinos, including women with GDM, youth and adults; and finalize with a discussion on lessons learned and opportunities to enhance research, and, consequently, clinical care oriented toward preventing type 2 DM in Hispanics/Latinos in the US and Latin America.

## Introduction

Poverty is the best cure for diabetes.

La pobreza es el mejor remedio contra la diabetes.

-Gabriel García Márquez (1)

Diabetes mellitus (DM) has been documented in the medical history of some Latin American countries since the late 1800s and early 1900s (2, 3). Although not a common disease at that time, type 1 DM was highlighted by medical societies in the Americas due to its dramatic presentation and fast clinical deterioration. In contrast, type 2 diabetes mellitus (type 2 DM) and its long-term complications only gained attention in the mid-twentieth century (2, 3). Over the next decades, type 2 DM would become recognized not just as a common chronic disease but also as one of the leading causes of death worldwide (4).

### Why Is It Important to Understand Type 2 Diabetes in Hispanics/Latinos in the US and in Latin Americans?

Hispanics/Latinos in the US mainland constitute 17.6% of the population (5), and are the largest US minority group. It is projected that by year 2060, 29% of the US population will be of Hispanic/Latino descent (6). Compared to other US populations, the Hispanic/Latino population is younger, with nearly half of the US-born Hispanics/Latinos under age 18 (7). The prevalence of diabetes among Hispanics/Latinos in the US is 22.6%, and twice as high as that of non-Hispanic Whites (NHWs) (11.3%) (8). Diabetes has also become a leading cause of death in Latin America (9, 10).

Understanding type 2 DM among Hispanics/Latinos in the US and in Latin America poses several challenges. A long-standing assumption in the US is that Hispanics/Latinos are a demographically and ancestrally homogeneous group. However, as we will explain, Hispanics/Latinos are a highly heterogeneous group, and in fact, the prevalence of diabetes varies widely among different US Hispanic/Latino heritage groups (11–13). These differences in prevalence may be mediated by differences in ancestry, as well as differences in socioeconomic factors, cultural norms, dietary and physical activity (PA) patterns, and history of environmental exposures—among others—experienced in both the country of origin and the US.

As the foundations for precision and personalized medicine are built, it is clear that adequate prevention, diagnosis, and treatment of type 2 DM among Hispanics/Latinos in the US and in Latin America require deep understanding of its etiology, manifestations, and response to interventions across populations. Whereas diabetes has become a seemingly unavoidable outcome for many Hispanics/Latinos, it does not have to be so.

In this expert review, we will describe the epidemiology of type 2 DM and gestational diabetes mellitus (GDM) and traditional cardiovascular (CV) risk factors in Hispanics/Latinos in the US and Latin America based on selected studies. We will also discuss studies on the etiology of type 2 DM and GDM in Hispanic/Latino populations. Given the increasing prevalence of type 2 DM across the continent, and especially at younger ages, we considered imperative to focus on research dedicated to disease prevention in Hispanics/Latinos in the US and in Latin America. We will also highlight opportunities to enhance our knowledge through research within a holistic framework of multidimensional and transdisciplinary strategies; a framework that is also applicable to the clinical care of Hispanics/Latinos at risk of type 2 DM.

In this review, the term type 2 DM will be used throughout most of the text, and specially within the context of etiology and prevention. The term diabetes will be used within the context of epidemiology or when there is no distinction between type 1 and type 2 DM. As a reference, we have also included a list of acronyms and abbreviations used throughout the text (see Appendix).

### Who Are the Hispanics/Latinos in the US and Latin Americans?

**Figure 1:**
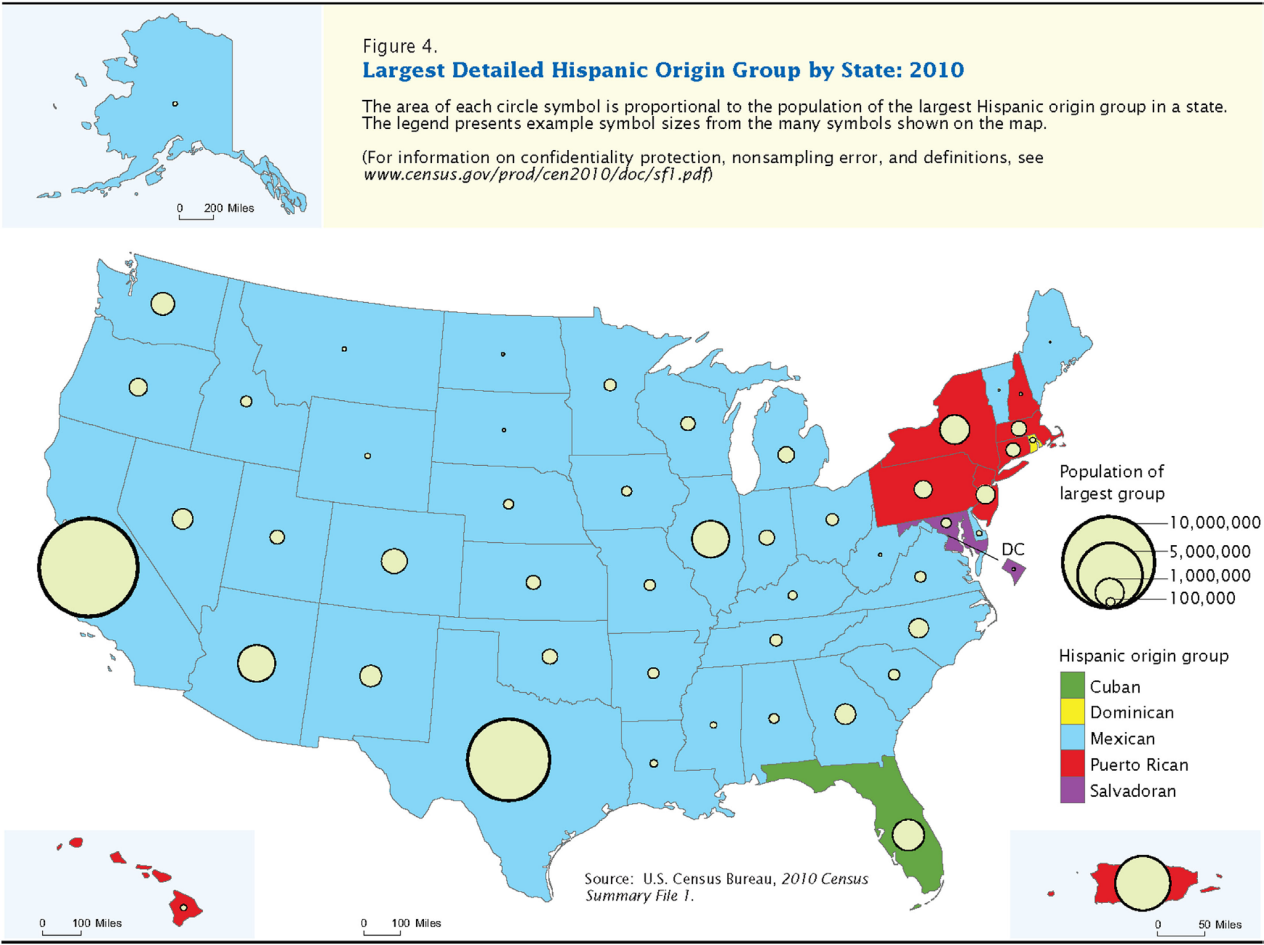
2010 US Census Map—Largest Detailed Hispanic Origin Group by State: 2010. This is Figure 4 from Ennis et al. (16).

The Office of Management and Budget (OMB) and the US Census Bureau define Hispanic or Latino as “a person of Cuban, Mexican, Puerto Rican, South or Central American, or other Spanish culture regardless of race” (14). The racial and ethnic categories defined by the OMB have been challenged and are under revision (15). Figure 1 (16) illustrates where the largest Hispanic/Latino heritage groups live in the US and Puerto Rico based on the 2010 US Census.

The terms Hispanic and Latino have different meanings in the US and abroad. Place of residence (17), ancestry group (18), or immigrant generation (18) may influence individuals’ preference for the term Hispanic, Latino, both, or neither. In this review, the term Hispanic/Latino refers to persons of Latin American descent who live in the US or in Latin America; that is, those who self-identify or trace their roots to countries in the Americas where Spanish, Portuguese, and French are the predominant languages (19, 20), excluding persons from Spain. The term Latin Americans refers to persons or populations in Latin America (19, 20).

### Sociodemographic and Economic Characteristics of the Hispanic/Latino Population in the US and in Latin America

According to the US Census Bureau, Hispanics/Latinos have become the US largest minority group. This population increased nearly fourfold between 1980 and 2015, rising from 14.8 million (6.5%) to 56.6 million (17.6%) (21–23). The Hispanic/Latino population is projected to increase to 119 million, or nearly 29% of the population in 2060 (6), with growth attributed to high rates of immigration and fertility, and low overall mortality (24). In 2015, Hispanics/Latinos of Mexican origin were the largest Hispanic/Latino heritage group (63%), followed by Puerto Ricans (10%), Salvadorans (4%), Cubans (4%), and Dominicans (3%) (21).

As a group, Hispanics/Latinos are younger than other racial/ethnic groups in the US (median 28.7 years) and lag behind other groups in educational attainment (21) (Table 1). At 67%, the percentage of Hispanics/Latinos age 25 or older with a high school education or higher is significantly lower than that of NHWs (91.8%) (25). Poverty rates are significantly higher among Hispanics/Latinos (21.4%) than NHWs (9.1%) (26). In the US, unemployment among the population aged 16 years and older was highest among non-Hispanic Blacks (11.3%) and islander Puerto Ricans (8.4%), and lowest among NHWs (5%) and Hispanics/Latinos (4.9%). Islander Puerto Ricans continue to have higher poverty rates (46.1%) than other Hispanics/Latino groups in the US (22.6%), non-Hispanic Blacks (25.4%), and NHWs (10.4%) (21).

**Table 1:** Selected socioeconomic indicators for Hispanics/Latinos in the US by heritage group, 2015.^a^

Race/ethnic group	Median age (years)	Percent of population ≥25 years who are high school graduate or higher	Percent of population ≥16 years who is unemployed	Percent of people whose income in the past 12 months is below the poverty level	Median family income	Percent of civilian noninstitutionalized population with no health insurance coverage
**US Mainland**						
Hispanic/Latino	28.7	66.0	4.9	22.6	$46,690	19.5
Mexican	26.9	60.9	4.9	23.5	$45,616	21.5
Puerto Rican	29.5	78.9	5.9	24.6	$45,693	8.5
Cuban	40.8	79.2	3.9	17.6	$51,105	13.9
Dominican	30.1	68.1	6.4	26.7	$39,762	12.3
Central American	30.1	56.0	4.6	22.2	$43,570	28.2
South American	35.6	85.2	4.3	14.4	$60,169	17.0
Other	31.7	70.8	4.7	20.0	$49,812	19.5
Non-Hispanic White	43.3	92.3	5.0	10.4	$77,072	6.3
Non-Hispanic Black	33.7	84.7	11.3	25.4	$45,014	11.0
**Puerto Rico**	40.0	74.7	8.4	46.1	$22,428	5.7

*^a^Source: US Census Bureau, 2015 American Community Survey 1-Year Estimates*.

Hispanics/Latinos of any race are disproportionately affected by lack of health insurance compared to all other groups (27). While only approximately 10% of NHWs did not have healthcare insurance, nearly 20% of Hispanics/Latinos of any race lack health insurance. Within Hispanic/Latino subgroups, the percentage of uninsured individuals varied considerably, from 8.5% in Puerto Ricans to 28.2% in Central Americans (28, 29).

The population in Latin America and the Caribbean is projected to grow from 646 million currently to 784 million in 2050 (30). This population is relatively young with a median age of 29.2 years (31) (Table 2). Enrollment in secondary and tertiary education remains unequal in the region, ranging from 21.2% in Honduras to 88.6% in Chile. The percentage of the population classified as poor is highly variable, from 4.3% in Argentina to over 60% in Guatemala and Honduras.

**Table 2:** Selected socioeconomic indicators for Hispanics/Latinos in Latin America, 2014–2015.

Country	Median age (years), 2015^a^	Gross enrollment rate in secondary and tertiary education, 2014^b^	Economic participation rate in population aged ≥ 15, 2015^b^	Percentage of population classified as poor, 2014^b^
Argentina	30.8	88.2/NA	59.6	4.3
Bolivia	24.1	75.7/NA	69.6	32.7
Brazil	31.3	NA	66.6	16.5
Chile	34.4	88.3/88.6	57.4	7.8
Colombia	30.0	78.6/51.3	69.4	28.6
Costa Rica	31.4	78.1/53.0	59.6	18.6
Cuba	41.2	NA/41.0	NA	NA
Dominican Republic	26.1	65.5/47.5	58.8	37.2
Ecuador	26.6	NA	64.4	29.8
El Salvador	26.7	70.2/28.9	61.6	41.6
Guatemala	21.2	46.7/NA	62.2	67.7
Honduras	23.4	49.3/21.2	61.6	74.3
México	27.4	NA/29.9	63.2	41.2
Nicaragua	25.2	NA	62.4	58.3
Panama	28.7	NA	64.0	21.4
Paraguay	24.9	NA	68.6	42.3
Perú	27.5	78.4/NA	71.8	22.7
Uruguay	34.9	NA	65.8	4.4
Venezuela	27.4	74.8/NA	64.8	32.1

*^a^Source: United Nations, Department of Economic and Social Affairs, Population Division (2015). World Population Prospects: The 2015 Revision, Key Findings and Advance Tables. Working Paper No. ESA/P/WP.241*.

*^b^Source: Economic Commission for Latin America and the Caribbean (ECLAC), Statistical Yearbook for Latin America and the Caribbean, 2016 (LC/PUB.2017/2-P), Santiago, 2016. Data correspond to the most recent available year*.

### Challenges in Identifying Hispanics/Latinos in US Government Reports and the Scientific Literature

#### Demographic Terminology

From the late 1800s and through most of the twentieth century, several terms have been used in the Census data and the medical literature to attempt to identify persons of Mexican origin, and later other Hispanics/Latinos (32, 33). Not surprisingly, the terms failed to accurately identify this elusive and complex mosaic of populations.

Through several modifications to the US Census since 1970 (33), blanket demographic terms such as “Hispanic” and “Latino” have been used to attempt to capture the specific cultural identities within the “Hispanic or Latino” populations. Despite the population’s diversity in virtually every element, this conglomerate of races, nationalities, and cultures is still regarded by many as a monolith.

#### Publications on US Hispanic/Latino Health

The earliest scientific reports on the health of any US Hispanic/Latino heritage group date from 1929 or earlier (34), and focused on tuberculosis in Mexican immigrants. Subsequent studies published from the 1950s to the 1970s addressed farm laborers’ health (35), folk medicine (36), sociological, psychiatric, and behavioral issues (37–42), hematologic and gastrointestinal disorders (43–46), infectious and tropical diseases (47–49), and women’s fertility (50), among other topics, and focused on Mexican immigrants, Puerto Rican migrants, and Puerto Rico. Thus, for most of the twentieth century, health-related studies in Hispanics/Latinos were isolated and did not address chronic diseases; furthermore, the manner in which Hispanics/Latinos were classified was inconsistent. Although the Framingham Heart Study was initiated in 1948 (51), and its first analyses on the incidence of cardiovascular disease (CVD) and associated risk factors were published between 1957 and 1959 (52, 53), the study of chronic diseases, in particular CV morbidity and mortality, in Hispanics/Latinos in the US did not start until the 1960s (54).

As we will describe, differences and similarities in the prevalence of diabetes and CV risk factors between Hispanics/Latinos and other racial/ethnic groups, and among Hispanic/Latino heritage groups would gradually be documented by various studies across the US. Understanding these differences and similarities could shed light on the interaction among various health determinants, and diabetes risk across populations. These differences and similarities are still being investigated.

## Epidemiology of DM in Hispanics/Latinos in the US and Latin America

### Diabetes in Hispanics/Latinos in the US

During the past three decades of the twentieth century, reports about chronic diseases—including diabetes—in the US Hispanic/Latino population started to be generated and disseminated through several epidemiological studies, including but not limited to the Puerto Rico Heart Health Program (54, 55), the Laredo Project (56), the San Antonio Heart Study (SAHS) (57, 58), the Hispanic Health and Nutrition Examination Survey (Hispanic HANES) (11, 59, 60), the San Luis Valley Diabetes Study (61), and later the National Health Interview Survey (NHIS) (28), the Behavioral Risk Factor Surveillance Survey (BRFSS) (62), and the National Health and Nutrition Examination Survey (NHANES) (63). However, most of the studies were cross-sectional and focused on one Hispanic/Latino heritage group or specific region of the US. Of these, the Hispanic HANES, conducted from 1982 to 1984, was the first epidemiological study to describe the prevalence of some chronic diseases, including diabetes, in the three largest Hispanic/Latino heritage groups at the time: Mexicans, Puerto Ricans, and Cubans (11, 59, 60). However, the Hispanic HANES was a one-time cross-sectional study. In 2006, the Hispanic Community Health Study/Study of Latinos (HCHS/SOL), a prospective study, was established to describe the prevalence of chronic diseases (especially CV and pulmonary), identify their risk or protective factors, and correlate them with future health outcomes in a cohort of US Hispanics/Latinos from diverse origins in a cohort of over 16,000 Hispanic/Latino adults from Central American, Cuban, Dominican, Mexican, Puerto Rican, and South American heritage living in San Diego, Chicago, Miami, and New York (64, 65).

There are important differences in these studies that make comparisons across them difficult: methodological variations in selection of study populations, diagnostic criteria to determine diabetes, lack of assessment to distinguish between type 1 and type 2 DM, proposed diagnostic cut points (66–68), and time-period of analysis. Further caution must be taken when interpreting data gathered across studies, because the lack of confirmatory testing of diagnostic criteria in some studies could result in an inaccurate estimate of the prevalence (66). Since some surveys restrict their sampling to specific geographic areas, generalizability of the findings to all Hispanics/Latinos living in the US or Latin America is reduced.

Table 3 lists selected US epidemiological studies focused on or that have included Hispanics/Latinos, and that have estimated diabetes prevalence (8, 11–13, 54–61, 64, 65, 69–95). An analysis of the 2011–2012 NHANES cycle showed that the unadjusted prevalence of total diabetes based on ADA criteria (2-h OGTT, FPG, or HbA1c) was 14.3% (9.1% for diagnosed diabetes and 5.2% for undiagnosed diabetes) and 38.0% for prediabetes (8). The prevalence of total diabetes for those of Hispanic/Latino heritage was twice as high (22.6%) than for NHWs (11.3%) and higher than the other racial/ethnic groups included in the study. The age-standardized prevalence of undiagnosed diabetes among Hispanic/Latino adults (49.0%) was significantly higher than that for NHWs (32.3%), and slightly lower than that for non-Hispanic Asians (50.9%) (8).

**Table 3:** Prevalence of type 2 diabetes and prediabetes among Hispanic/Latino populations in the US (selected studies).

Study (reference)	Study period	Sample size	Age range (years)	Glycemic criteria	Key findings
Puerto Rico Heart Health Program (54, 55)	1965–1980	2,567 rural and 6,190 urban Puerto Rican men	45–64	FPG ≥ 126 mg/dL, self-reports of physician-diagnosed diabetes, or use of insulin or hypoglycemic agents	3.6% in rural men and 9% in urban men
Laredo Project (56)	1979	389 Mexican Americans	40–74	Fasting hyperglycemia defined as FPG > 140 mg/dL	Age-adjusted prevalence was 10.9% in men and 10.1% in women
Starr County Study, Texas (69)	1981	2,498 Mexican Americans	≥15	Zero-hour (fasting) plasma glucose level ≥140 mg/dL or a 2-h level ≥200 mg/dL.	6.9% males and 6.7% females
Albuquerque, New Mexico (70)	1984–1985	1,175 Hispanics	≥18	Self-reported diagnosis	Prevalence increased with age: men: 1.9% in 25–34 to 13.8% in ≥75. Women: 0.6% in 25–34 to 16.1% in ≥75
San Luis Valley Diabetes Study, Colorado (61, 71)	1984	607 Hispanics without prior history of DM 343 Hispanics with previously diagnosed DM	20–74	History of previously diagnosed DM with confirmation using FPG ≥ 140 mg/dL or 2-h OGTT ≥ 200 mg/dL	Age-adjusted prevalence of previously diagnosed DM: 3.3% in men and 4.9% in women Newly diagnosed DM: 2.2% in men and 4.3% in women
San Antonio Heart Study, Texas (57, 58, 72)	First phase: 1979–1982 Second phase: 1984–1988	3,302 Mexican Americans	25–64	2-h OGTT	Age-adjusted prevalence for men was 14.0% in the barrio vs. 6.5% in the suburbs and for men was 18.0% in the barrio vs. 4.3% in the suburbs. Prevalence was two to three times higher in Mexican Americans than in NHWs.
Puerto Rico Household Health Interview Survey (73)	1975–1986	Minimum sample size of 2,696 in 1983 Maximum sample size of 12,212 in 1976	All ages	Self-reported diagnosis	3.1% in 1975 to 5.1% in 1986 4.5% of males and 5.8% of females reported a history of DM
HHANES (11)	1982–1984	6,588 Hispanics	20–74 years	OGTT administered to subsample of 1,326	Cubans 45–74: 15.8% Mexican Americans 45–74: 23.9% Puerto Ricans 45–74: 26.1% Cubans 20–44: 2.4% Mexican Americans 20–44: 3.8% Puerto Ricans 20–44: 4.1%
Mexico City Diabetes Study (74)	1979–1982	2,282 individuals of Mexican descent in San Antonio, and Mexicans in Mexico City	35–64	FPG ≥ 126 mg/dL	San Antonio: 17.8% in men and 23% in women Mexico City: 12.3% in men and 18.5% in women
NHANES (75)	1999–2000	915 Mexican Americans, NHBs, and NHWs	12–19	IFG: 100–125 mg/dL	Prevalence of IFG was 13.0, 4.2, and 7% in Mexican Americans, NHBs, and NHWs, respectively
NHANES (76)	1999–2002	1,802 adolescents without DM (672 Mexican American)	12–19	HOMA-IR	Mexican American children had higher HOMA-IR levels than white children
NHANES (77)	1988–1994 1999–2002	1988–1994: 31,638 Mexican American, NHW and NHB adults 1999–2002: 17,217 Mexican American, NHW and NHB adults	≥20	Previously diagnosed DM Undiagnosed DM: FPG ≥ 126 mg/dL IFG: 100–125 mg/dL	Among Mexican Americans: Previously diagnosed DM: 9.6 and 10.6% Undiagnosed DM: 4.7 and 3.5% IFG: 32.9 and 33.0%
MESA (78)	2000–2002	1,437 Hispanics	45–84	FPG ≥ 126 mg/dL or use of hypoglycemic medication	Age-adjusted prevalence of diabetes Mexican Americans: 22.3% Dominicans: 16.1% Puerto Ricans: 19.7% Other Hispanics: 15.2%
NHANES (79)	2005–2006	2,806 Mexican American, NHW and NHB adults	≥20	FPG, OGTT	Mexican Americans Diagnosed DM: 12.6% Undiagnosed DM: 7.5% Total DM: 20.1% Percent undiagnosed DM: 35.9% Prediabetes: 32.0% Prediabetes/DM: 52.0%
NHANES (80)	2005–2006	277 Mexican Americans 257 NHBs 198 NHWs	12–19	FPG and 2-h OGTT ADA 1998	Prevalence of IFG, IFT, and prediabetes among Mexican Americans were 14.3, 3.5, and 16.9%, respectively
Study of Women’s Health Across the Nation (SWAN) (81)	1996–1997	3,302 women in seven sites; analysis based on 420 women recruited in the New Jersey site	42–52	FPG ≥ 126 mg/dL or self-reported use of insulin	3.3% in Caucasians, 5.9% in Dominicans, 8.3% in Central Americans, 9.8% in Cubans, 13.5% in Puerto Ricans, 15.6% in South Americans
Phase I of the US–Mexico Border Diabetes Prevention and Control Project (82)	2001–2002	4,027: 2,120 Hispanics from Mexico, 1,437 Hispanics from the US, and 470 non-Hispanics from the US.	≥18	Self-reported diagnosis FPG ≥ 126 mg/dL	Age-adjusted prevalence of self-reported and unrecognized DM was 16.6% of the Mexican side and 14.7% on the US side; age-adjusted prevalence of IFG was similar on both sides of the border (14.1% on the Mexican side and 13.6% on the US side)
BRFSS (83)	1995–2010	1995: 1,193 in Montana to 5,107 in Maryland 2010: 1,964 in Alaska to 35,109 in Florida	≥18	Self-reported diagnosis	In 1995, age-adjusted prevalence was >6% in only three states, Washington, DC, and Puerto Rico. In 2010, age-adjusted prevalence was highest (>10%) in Alabama, Mississippi, Puerto Rico, South Carolina, Tennessee, Texas, and West Virginia.
NHANES (85)	1999–2008	22,621 Mexican American, NHW and NHB adults, of whom 551 had undiagnosed DM	≥20	Undiagnosed DM was defined as HbA1c ≥ 6.5% without a self-report of physician-diagnosed DM	Overall prevalence of undiagnosed DM of 1.5% in NHWs, 3.1% in NHBs, and 2.7% in Mexican Americans Overall prevalence of diagnosed DM of 6.7% in NHWs, 11.3% in NHBs, and 7.6% in Mexican Americans
California Health Interview Survey (86)	2001, 2003, 2005, and 2007	33,633 Hispanics and 126,488 NHWs	≥18	Self-report of physician-diagnosed DM	Mexicans: 9% Central Americans: 1% South Americans: 0.5% 2 or more countries of origin: 7% Other Hispanics: 13% NHWs: 7%
NHANES (87)	1999–2002 2003–2006 2007–2010	19,182 Mexican American, NHW and NHB adults	≥12	HbA1c: 5.7–6.4% FPG: 100–125 mg/dL	Prevalence of prediabetes among Mexican Americans: 34.4, 30.3, and 37.8%
HCHS/SOL (12, 84)	2008–2011	16,415 Hispanics	18–74	FPG, 2-h OGTT, HbA1c	Overall prevalence of DM was 16.9% South Americans: 10.2% Cubans: 13.4% Central Americans: 17.7% Dominicans: 18.1% Puerto Ricans: 18.1% Mexicans/Mexican Americans: 18.3%
SEARCH Study (88, 89)	2001–2009	3,345,783 youths in 2001 and 3,458,974 in 2009	<20	Physician-diagnosed DM	Prevalence of type 2 DM among Hispanic youth aged 10–19 years was 0.45 per 1,000 in 2001 and 0.79 per 1,000 in 2009, representing a relative increase of 76% over the 8-year period
NHANES (90)	1988–1994 1999–2004 2005–2010	1988–1994: 4,201 1999–2004: 2,860 2005–2010: 2,765 Mexican Americans	≥20	Based on HbA1c	Diagnosed DM: 7.6 (0.57), 8.8 (1.06), 11.9 (1.01) Undiagnosed DM: 1.8 (0.17), 2.3 (0.40), 2.9 (0.32) Prediabetes: 5.6 (0.64), 10.6 (0.68), 12.7 (0.76)
NHANES (8)	1988–2012	2011–2012: 2,623 (561 All Hispanics)	≥20	Self-reported diagnosis Undiagnosed DM based on FPG, 2-h OGTT, HbA1c	All Hispanics Total DM: 22.6% Diagnosed DM: 12.5% Undiagnosed DM: 10.1% Prediabetes: 36.8% Mexican Americans Total DM: 23.8% Diagnosed DM: 14.4% Undiagnosed DM: 9.4% Prediabetes: 38.0%
NHIS (91)	1997–2012	427,975 (367,292 non-Hispanics and 60,683 Hispanics)	≥18	Self-reported diagnosis	Less than high school education: 17.6% for Puerto Ricans, 13.4% for Cubans, and 9.7% for Mexicans High school diploma/GED: 9.8% for Puerto Ricans, 8.2% for Cubans, and 6.3% for Mexicans More than high school education: 6.8% for Puerto Ricans, 6.0% for Cubans, and 6.7% for Mexicans
HCHS/SOL (13)	2008–2011	15,507 Hispanics	18–74	FPG, 2-h OGTT, HbA1c	6.7% met at least one diagnostic criterion of probable DM – 39.4% of total DM (self-reported plus probable DM)
HCHS/SOL (92)	2012–2014	1,466 Hispanics	8–16	FPG and HbA1c ADA 2015	Prevalence of prediabetes/DM was 16.5%, being higher in boys (20.9%) than in girls (11.8%)
Starr County, Texas (93)	2002–2014	5,230 Mexican American adults	≥20	FPG, OGTT, HbA1c	Previously identified DM: 11.8% in men and 14.4% in women Newly identified DM: 11.9% in men and 12.3% in women Total DM: 23.7% in men and 26.7% in women Prediabetes: 32.8% in men and 31.9% in women
NHANES (94)	2005–2014	2,606 NHWs, NHBs, and Hispanics	12–19	FPG, 2-h OGTT, HbA1c	Prevalence of total DM, prediabetes, and percent undiagnosed DM: All Hispanics: 0.76, 22.9, and 39.5% Mexican Americans: 0.85, 22.4, and 43.9%
HCHS/SOL (95)	2008–2011	15,507 Hispanics	18–74	FPG, 2-h OGTT, HbA1c	Prediabetes: 36.3% Total IFG: 18.1% Total IGT: 15.4% Total impaired HbA1c: 21.2% IFG + impaired HbA1c: 8.6% IFG + IGT + impaired HbA1c: 3.8%

The baseline examination of the HCHS/SOL (2008–2011) showed that the prevalence of diabetes ranged from 10.2% in South Americans to 18.1% in Dominicans and Puerto Ricans and 18.3% in Mexicans (12). Prevalence by sex ranged from a low of 10.6% for South American men to a high of 18.7% for Mexican men, and from 9.8% in South American women to 19.5% in Puerto Rican women. Diabetes prevalence increased significantly with age and BMI but was inversely associated with years of education and household income (12). Although the prevalence of diabetes also increased significantly with length of residence in the US, it was similar among US-born participants and foreign-born participants residing in the US for fewer than 5 years. However, the prevalence was significantly higher among foreign-born participants with longer than 10 years of residence in the US. Nearly half of Hispanics/Latinos (48%) had adequate glycemic control, a rate slightly lower than those for NHWs (52.9%) and non-Hispanic Blacks (52.6%) who participated in the 1988–2010 NHANES cycles (96). Preliminary data from the second examination of the HCHS/SOL (2014–2017) demonstrated an overall increase in prevalence of diabetes, which is still higher among Mexicans, Dominicans, and Puerto Ricans and lower among Cubans (97).

The HCHS/SOL also determined that nearly 40% of Hispanics/Latinos with diabetes met at least one ADA criterion of undiagnosed diabetes (13). This finding is consistent with a recent NHANES data analysis that showed that Mexican Americans and other Hispanic women were more likely to be unaware of their diabetes status (98).

An additional analysis of HCHS/SOL baseline data found that 36.3% of the target population met at least one of the ADA criteria for prediabetes (97). Prevalence of prediabetes varied by age, sex, Hispanic/Latino heritage groups, and BMI categories, but was similar across place of birth and years living in the US. In the 2011–2012 NHANES cycle, the overall prevalence of prediabetes was 38.0%, and 36.8% for all Hispanics/Latinos (8).

Although there is a paucity of studies examining the prevalence of type 2 DM among children and adolescents, evidence suggests that prediabetes is highly prevalent among Hispanic/Latino adolescents in the US. In a recent analysis based on the 2005–2014 NHANES cycles, the prevalence of prediabetes (based on FPG, 2hPG, or HbA1c) among all Hispanic/Latino adolescents (aged 12–19 years) was 22.9%. Among Mexican Americans it was 22.4%, significantly higher (*P* = 0.001) than in NHWs (15.1%) (94). Recent data from the Study of Latino Youth, a population-based cross-sectional study of 1,466 Hispanic/Latino youth aged 8–16 years, found a combined prevalence of prediabetes and diabetes (based on FPG and HbA1c) of 16.5% (92), with boys having a higher prevalence compared with girls (20.9 vs. 11.8%, respectively). However, the study did not assess differences by Hispanic/Latino heritage group nor was OGTT available.

The limited number of studies examining the incidence of type 2 DM in Hispanic/Latinos (99–106) indicates that the incidence has been higher than in NHWs across all age groups (99–101) (Table 4). A recent analysis of type 2 DM in children examined in five study centers in the U.S (105) reported an annual rate increase significantly greater for Hispanics/Latinos (3.1%) compared to NHWs (0.6%), but significantly lower than Native Americans (8.9%). However, a recent analysis based on NHANES 2005–2014 cycle did not reveal an increase in incidence in this age group (94).

**Table 4:** Incidence of type 2 diabetes among Hispanic/Latino populations in the US (selected studies).

Study and reference	Study period	Sample size	Age range (years)	Glycemic criteria	Key findings
San Antonio Heart Study, Texas (99)	1979–1992	3,302 Mexican Americans	25–64	2-h OGTT	8-year incidence was 8.7% among Mexican Americans in the low-income barrio neighborhoods; 8.4% among Mexican Americans in the transitional neighborhoods; and 3.4% among Mexican Americans in the suburban neighborhoods
San Luis Valley Diabetes Study, Colorado (100)	1983–1988	607 Hispanics without prior history of DM 343 Hispanics with previously diagnosed DM	20–74	History of previously diagnosed DM with confirmation using FPG ≥ 140 mg/dL or 2-h OGTT ≥ 200 mg/dL	6-year incidence for Hispanic men and women was 4.3/1,000 and 5.3/1,000, respectively; incidence was 2.4 (95% CI: 1.6–3.6) times higher in Hispanic men than in NHW men; incidence in Hispanic women was 3.6 (95% CI: 2.4–5.4) times higher.
San Antonio Heart Study, Texas (101)	1987–1996	1,995 Mexican Americans 1,231 NHWs	25–64	2-h OGTT ≥ 200 mg/dL FPG ≥ 126 mg/dL History of DM and receiving treatment with insulin or oral hypoglycemic agents	7- to 8-year incidence increased from 5.7% for Mexican Americans enrolled in 1979 to 15.7% for participants enrolled in 1988.
San Antonio Heart Study, and Mexico City Diabetes Study (102)	1987–1996	1,995 Mexican Americans 1,231 NHWs	35–64	2-h OGTT ≥ 200 mg/dL FPG ≥ 126 mg/dL History of DM and receiving treatment with insulin or oral hypoglycemic agents	6- to 8-year crude incidence was 90% higher in Mexican Americans men living in San Antonio than Mexicans in Mexico City: 2.70/100 versus 1.42/100; in women, the crude incidence was more than twofold higher in San Antonio than in Mexico City: 2.86/100 versus 1.21/100. Incidence remained significantly higher in San Antonio than in Mexico City after adjusting for several confounders.
SEARCH for Diabetes in Youth Study, US (103)	2002–2005	635 incident cases from 3,207,005 Hispanic person-years at risk	<20	Physician-diagnosed DM	Incidence of type 1 DM for youth aged ≤ 14 years was 15.0/100,000 for females and 16.2/100,000 for males. Among youth aged ≤ 17 years, the rates of type 1 DM were 14.1/100,000 for females and 15.6/100,000 for males. For type 2 DM, the rates were 6.9/100,000 for females and 4.8/100,000 for males.
Puerto Rico Heart Health Program (104)	1965–1975	4,685 Puerto Ricans	35–79	FPG ≥ 126 mg/dL and self-reports of physician-diagnosed diabetes or use of insulin or hypoglycemic agents	Crude rate of 11% over a median follow-up period of 2.6 years (range: 2–7 years), for an incidence rate of 42.6/1,000 person-years.
New York City Community Health Survey (105)	2002, 2004, and 2008	24,384	≥18	Self-reported DM	Age-adjusted incidence of DM per 1,000 population was 9.4 in 2002, 11.9 in 2004, and 8.6 in 2008. DM incidence was significantly associated with being aged 45 or older, being Black or Hispanic, being overweight or obese, and having less than a high school diploma.
SEARCH for Diabetes in Youth Study, US (106)	2002–2012	11,245 youths aged < 20 with type 1 DM and 2,846 youth aged 10–19 with type 2 diabetes	<20	Physician-diagnosed DM	Annual rate of increase in type 1 DM was greater among Hispanics than among NHWs (4.2 vs. 1.2%, *P* < 0.001). Annual rate of increase in type 2 DM among Hispanics differed significantly from that among Native Americans (3.1% vs. 8.9%, *P* = 0.01).

A few studies have estimated GDM in Hispanics/Latinos (107–114) (Table 5). One of the early studies examining the epidemiology of GDM in an ethnically diverse cohort of over 10,000 women at the Mount Sinai Medical Center in New York City between 1987 and 1990 found a prevalence of 3.2% (107). After controlling for traditional risk factors, first-generation Hispanic/Latino women had a 59% increased risk for GDM compared to NHW women. Kim et al. (112) found that among Hispanic/Latino women, the prevalence of GDM varied by heritage group, from 5.5% among Cubans to 8.7% among Mexicans. Nearly 45% of GDM cases among Hispanic/Latino women were attributable to overweight and obesity, which ranged from 38.5% among Central/South American women to 65.7% among Cuban women. In a recent study of prevalence trends in GDM by race/ethnicity for 19 states, the GDM prevalence increased significantly from 3.71 per 100 deliveries in 2000 to 5.77 per 100 deliveries in 2010 (113). Prevalence of GDM among deliveries increased significantly among all racial/ethnic groups, particularly Hispanics/Latinas, who experienced the highest relative increase (66%).

**Table 5:** Incidence or prevalence of gestational diabetes mellitus among Hispanic/Latino populations in the US.

Study and reference	Study period	Sample size	Age range (years)	Glycemic criteria	Key findings
New York City (107)	1987–1989	10,187 (328 with GDM; of these 112 Hispanic)	<20 to ≥40	FPG and OGTT according to National Diabetes Data Group	Prevalence of 4.1% among Hispanics Excess risk for Hispanic women born in Puerto Rico or elsewhere outside of the US
Nurses’ Health Study II (108)	1990–1994	14,613 (224 Hispanic)	25–42	Self-reported GDM, validated by medical record review in a subset	4.7% Whites, 10.6% AAs, 7.6% Hispanics, and 10.5% Asians
Northern California Kaiser Permanente Medical Care Program (109)	1991–2000	267,051 pregnancies screened for GDM	15–49	Physician diagnosis or ADA criteria	Age- and race/ethnicity-adjusted yearly prevalence increased from 5.1% in 1991 to 7.4% in 1997, being significantly higher among Asians (7.2–11.0%) and Hispanics (7.2–9.8%)
New York City (110)	1995–2003	951,920	15–44	Diagnostic codes ICD–9 648.81–648.82	Overall risk of 5.2% 7.2% North African 5.9% Sub-Saharan Africa 6.2% East Asia 8.6% South-East Asia and Pacific Islands 14.3% South Central Asia 6.8% Non-Hispanic Caribbean 4.9% Hispanic Caribbean 6.3% Mexico 4.9% Central American 6.6% South American 34.3% African-American 3.6% NHWs
19 US states (111)	2005–2006	3,108,877 births	<20 to ≥40	Documented in birth certificate	3.8% NHWs, 3.5% NHBs, 3.6% Hispanics, 6.3% Asian, and Pacific Islanders
California (112)	2007–2009	1,228,265 records	≥20	ICD-9-CM codes for pregestational DM (250) or DM (648.0) GDM was defined as glucose intolerance complicating pregnancy, childbirth, or postpartum (ICD-9-CM code 648.8).	11.9% Asian and Pacific Islands, 7.6% American Indians, 5.6% Black Americans, 8.4% Hispanics, 6.6% others, and 5.4% NHWs Among Hispanic women: Mexican (8.7%), Central/South American (7.4%), Puerto Rican (6.6%), and Cuban (5.5%)
GDM among hospital deliveries in 19 US states (113)	2000–2010	75,212 in 2000 to 119,229 in 2010	15–44	ICD-9-CM 648.8x, DRG codes 767–768, 774–775, and 765–766 (2008–2010), or DRG codes 370–375 (2000–2007)	Age-standardized prevalence increased from 3.71% in 2000 to 5.77% in 2010 Largest relative increase in prevalence was highest among Hispanics (66%)
Prepregnancy diabetes among deliveries in 19 US states (114)	2000–2010	13,217 in 2000 to 18,168 in 2010	15–44	ICD-9-CM 648.0x. 250.xx, or 249.xx or DRG codes 767–768, 774–775, and 765–766 (2007–2010) or DRG codes 372–375 (2000–2007)	Age-standardized prevalence of deliveries with prepregnancy DM among Hispanics ranged from 0.74 in 2000 to 0.94 in 2010 NHBs, followed by Hispanics, had the highest absolute change in age-standardized rates (0.20 per 100 deliveries) and throughout the time period had the highest rates of prepregnancy DM

*ICD-9, International Classification of Diseases, Ninth Version; ICD-9-CM, International Classification of Diseases, Ninth Version, Clinical Modification. DRG, Diagnosis-Related Group*.

### Diabetes in Latin America

Table 6 illustrates selected studies assessing diabetes in Latin America (115–139). The prevalence of diabetes varies by date in which the studies were performed, methods of assessing prevalence, region and age group (140, 141).

**Table 6:** Prevalence of type 2 diabetes and prediabetes among Hispanic/Latino populations in Latin America (selected studies).

Study and reference	Study period	Sample size	Age range (years)	Glycemic criteria	Key findings
The Brazilian Cooperative Group on the Study of Diabetes Prevalence (115)	1986–1988	21,847 from nine large cities	30–69	2-h OGTT WHO 1985	Overall prevalence was 7.6 and 7.8% for diabetes and IGT, respectively. DM prevalence increased from 2.7% in the 30–39-year age-group to 17.4% in the 60–69-year age-group
The Cooperative Group for the Study of Diabetes Prevalence in Rio de Janeiro, Brazil (116)	1988–1989	2,051	30–69	2-h OGTT WHO 1985	Age-adjusted prevalence rates for DM and IGT were 7.1 and 9.0%, respectively
Bolivia (117)	1998	2,948 from four Bolivian cities: La Paz, El Alto, Santa Cruz, and Cochabamba	≥25	2-h OGTT WHO 1997	Overall prevalence of DM was 7.2% and of IGT was 7.8%.
Brazilian Multicenter Study, Ribeirão Preto, São Paulo, Brazil (118)	1986–1988	1,473	30–69	2-h OGTT WHO 1997	The overall rates of DM and IGT were 12.1 and 7.7%, respectively
Mexican National Health Survey 2000 (119)	2000	42,886 from 400 cities in Mexico	≥20	2-h OGTT FBG ADA 1997	Age-adjusted prevalence 8.2%, of which 2,878 (80%) had previously been diagnosed.
Argentina (120)	1995–1998	2,397	≥20	2-h OGTT WHO 1999	Age-standardized prevalence rates for diabetes ranged between 6.5 and 7.7%
Mexican National Health Survey 2000 (121)	2000	12,856 men and 28,332 women	20–69	2-h OGTT FBG ADA 1997	DM rates of 5.6% in men and 9.7% in women
The Health Wellbeing and Aging (SABE) Project and NHANES (123)	1999–2004	10,518	≥60	Self-reported diagnosis	13.0% in Santiago, 13.8% in Montevideo, 14.7% in Havana, 17.7% in São Paulo, 21.3% in Mexico City, 21.7% in Bridgetown US Whites 13.8% US Blacks 25.5% US Mexican Americans 26.4%
The Health Wellbeing and Aging (SABE) Project (124)	1999–2000	13,753	≥60	Self-reported diagnosis	Prevalence of diagnosed diabetes was highest in US Blacks and Mexican Americans, followed by Bridgetown and Mexico City (22% for each) and lowest in Santiago, Montevideo, Havana, and US Whites (13–15%).
The FRIMEX Study (125)	2001–2002	140,017 from 6 Mexican cities: Mexico City, Guadalajara, Monterrey, Puebla, Leon, and Tijuana	≥18	FBG > 126 mg/dL Random > 200 mg/dL	Prevalence of 10.4%
PREVENCION Peruvian Study of the Prevalence of Cardiovascular Diseases (126)	2004–2006	1,878 adults in Arequipa, Peru	20–80	FPG ≥ 100 mg/dL or current antidiabetic medication (insulin or oral agents)	Age-standardized prevalence was 6.3% in men and 5.9% in women
Pelotas, Rio Grande do Sul State, Brazil (127)	2003	3,100 men and women	≥20	Self-reported diagnosis	Prevalence of DM of 6.3%
CARMELA Study (128)	2003–2005	11,550 in 7 Latin American countries	25–64	FPG ≥ 126 mg/dL or self-reported diagnosis	Prevalence of DM was 7% (range: 4–9%)
CARMELA Study (129)	2003–2005	11,502 in 7 Latin American countries	25–64	FPG ≥ 110 mg/dL or self-reported diagnosis	31.2% had glucose abnormalities (32.6% of men and 30.1% of women)
Mexico City Prospective Study (130)	1998–2004	52,584 men and 106,962 women	≥35	Self-reported diagnosis	4.8% in men and 3.9% in women
Chile (131)	2000–2005	486 overweight and obese adolescents	6–15	FPG ≥ 100 mg/dL	2% in males Tanner stage I-II 1% in females Tanner stage I-II 9% in males Tanner stage III-V 6% in females Tanner stage III-V
The Latin American Consortium of Studies in Obesity (LASO) (133)	1998–2007	31,009 Argentina, Chile, Colombia, Costa Rica, Dominican Republic, Perú, Puerto Rico, and Venezuela	20–85	FPG ≥ 126 mg/dL or self-reported current pharmacological treatment	Average prevalence of 5%, 4.8% in women and 5.1% in men Puerto Rico had a significantly higher prevalence of DM
Brazilian Longitudinal Study of Adult Health (ELSA-Brasil) (135)	2008–2010	15,105	35–74	Self-reported diagnosis, medication use, FPG ≥ 126 mg/dL, 2-h post-OGTT ≥ 200 mg/dL, or HbA1c ≥ 6.5%	Prevalence of DM was 19.7% Proportion of previously diagnosed DM was 50.4%
CESCAS [*Centro de Excelencia en Salud Cardiovascular para el Cono Sur*] I Study (136)	2010–2011	7,524 Argentina, Chile, and Uruguay	35–74	FPG ≥ 126 mg/dL or self-reported history of diabetes	Prevalence of DM of 12.4%, 10.6% in men, and 14% in women
Panama Survey of Life and Health Quality (ENSCAVI) and Survey of Risk Factors Associated with Cardiovascular Diseases (PREFREC) (137)	2007 2010–2011	25,748 3,590	≥18 ≥18	Self-reported diagnosis of diabetes FPG ≥ 126 mg/dL and HbA1c ≥ 6.5%	Prevalence of DM of 5.4% Prevalence of previously diagnosed DM of 7.3% and combined prevalence of DM of 9.5% based on FPG and HbA1c
Multiethnic Study of Pre-Diabetes and Diabetes in LMIC (138)	2010–2011	23,496 Southern Cone of Latin America, Peru, South Asia, and South Africa.	≥20	Diabetes: FPG ≥ 126 mg/dL Prediabetes: 100–125 mg/dL	Prevalence of DM and prediabetes was 14.0 and 17.8%, respectively, in the Southern Cone of Latin America; estimates were 9.8 and 17.1%, respectively, in Perú.
CRONICAS Centro de Excelencia de Enfermedades Crónicas Cohort Study (139)	2010–2013	3,135 Four Peruvian sites	≥35	FPG ≥ 126 mg/dL or self-reported current pharmacological treatment	Overall prevalence of 7.1%

*FBG, fasting blood glucose*.

According to a 2015 International Diabetes Federation report, the prevalence of type 2 DM in Latin America was highest in Mexico (14.7%) and lowest in Argentina (6.2%); however, over one-quarter of the Latin American and Caribbean population remained undiagnosed (141). The Cardiovascular Risk Factor Multiple Evaluation in Latin America (CARMELA) Study (conducted between 2003 and 2005) (128) reported an age-adjusted prevalence of diabetes of 7.0% in seven urban populations in Latin America (129). The prevalence was highest in Mexico City, Mexico (8.9%) and Bogotá, Colombia (8.1%) and lowest in Lima, Perú (4.4%); it increased with age in all cities, and tended to be higher among women than men. Prevalence of undiagnosed diabetes occurred in 20% of adults with diabetes. The Latin American Consortium of Studies in Obesity (LASO) (which included 11 studies conducted in eight countries between 1999 and 2008) (132) reported an average prevalence of 5%, which increased from 0.9% in participants aged 20–29 years to 16.4% in those aged 70 or older (133). However, the prevalence of diabetes in Latin America and the Caribbean was similar to the US population (NHANES, 1999–2004), after accounting for differences in the age distribution. Puerto Rico had the highest prevalence among the countries included in the analysis.

More recent Latin American studies include the *Estudo Longitudinal de Saúde do Adulto* (ELSA-Brasil) (134), which reported a prevalence of 19.7% (based on self-report or FPG, 2hPG and HbA1c), of which about half was undiagnosed (135); the *Centro de Excelencia en Salud Cardiovascular para el Cono Sur I* (CESCAS I), which reported an overall prevalence (based on FPG only) of 12.4% across four sites in Argentina, Chile, and Uruguay (136); and the *Centro de Excelencia de Enfermedades Crónicas* (CRONICAS), which reported an overall prevalence of 7.1% (based on FPG only) across four regions in Perú (139).

One of the few studies in Latin America and the Caribbean that estimated the incidence of type 2 DM is the Mexico City Diabetes Study, which focused on the population aged 35–64 years over 18 years of follow-up (143) (Table 7). Cumulative incidence rates of type 2 DM for men and women were 14.4/1,000 and 13.7/1,000, respectively. Incidence was 15.8, 15.7, and 12.7 per 1,000 person-years for the follow-up studies conducted in 1994, 1998, and 2008, respectively. These figures suggest that this population has one of the highest diabetes incidence rates worldwide, and that incidence rates have remained fairly stable.

**Table 7:** Incidence of type 2 diabetes and gestational diabetes mellitus among Hispanic/Latino populations in Latin America.

Study and reference	Study period	Sample size	Age range (years)	Glycemic criteria	Key findings
Brazilian Gestational Diabetes Study (142)	1991–1995	4,977	≥20 years	OGTT ADA 2000 WHO 1999	2.4% presented with GDM by ADA criteria and 7.2% by WHO criteria.
Mexico City Diabetes Study (143)	1990–2008	1,939	35–64	OGTT ADA criteria	Cumulative incidence was 14.4/1,000 and 13.7/1,000 for men and women, respectively. Incidence was 15.8/1,000, 15.7/1,000, and 12.7/1,000 for the second (1994), third (1998), and fourth (2008) follow-up phases, respectively.
CRONICAS Cohort Study (139)	2010–2013	3,135 Four Peruvian sites	≥35	FPG ≥ 126 mg/dL or self-reported current pharmacological treatment	Overall incidence of 1.95 per 100 person-years. 1.97 per 100 person-years in women and 1.93 per 100 person-years in men.

### Other CV Risk Factors in Hispanics/Latinos

#### Overweight/Obesity

In the most recent NHANES examination (2013–2014 cycle), the age-adjusted prevalence of obesity in adults (>20 years) was 37.1% for NHWs and 42.7% for Hispanics/Latinos, with Hispanic/Latino men and women having 21% and 33% increased odds of obesity compared to NHWs, respectively (144). In the most current NHANES examination, the prevalence of obesity in children was 14.7% for NHWs and 21.9% in Hispanics/Latinos, and extreme obesity was 4.4% in NHWs and 7.6% in Hispanics/Latinos (145). In the baseline examination (2008–2011) of the HCHS/SOL, 77% of participants had overweight or obesity, and the age-adjusted prevalence of obesity ranged from 26.8% in South American men to 51.4% in Puerto Rican women (84). However, enlarged waist circumference (≥102 cm in men and ≥88 cm in women) was highly prevalent among men (73%) and women (96%), independent of the heritage group (146). A higher prevalence of overall obesity in women compared to men, and high prevalence of abdominal obesity have also been described in Puerto Rico (147, 148).

The CARMELA study reported an overall obesity prevalence of 23%, ranging from 16% in Quito, Ecuador to 31% in Mexico City, Mexico (128). The LASO reported that overall obesity ranged from 13.8% in men to 18.4% in women, and that abdominal obesity ranged from 15.4% in men to 55.5% in women (133). CESCAS I reported a prevalence of 35.7% obesity and 52.9% central obesity (136), whereas CRONICAS reported a prevalence of 44.0% overweight and 26.0% obesity among individuals without diabetes, and 40.8% overweight and 39.9% obesity among those with diabetes; and enlarged waist circumference in 71.5% without diabetes and 88.5% with diabetes (139); and in ELSA-Brasil, 40.2 and 22.9% had overweight and obesity, respectively (149). Although the prevalence of obesity in some regions of Latin America has been lower than that of Hispanics/Latinos in the US, in others it is comparable.

A systematic review of studies published between 2008 and 2013 estimated that the prevalence of overweight and obesity for children and adolescents throughout Latin America ranged from 18.9 to 36.9% in children aged 5–11 and 16.6 to 35.8% in adolescents aged 12–19 (150). An obesity trend analysis from Mexico (2012) revealed a significant increase during the same time frame in which obesity increased in the US (145, 151). The prevalence of overweight/obesity also increased in Colombia from 38.2% in 2000 to 43.1% in 2010 (152).

#### Hypertension

In the 2009–2010 NHANES cycle, the age-adjusted prevalence of hypertension among US adults was 29.8% for NHW men, 26.9% for NHW women, 26.3% for Mexican American men, and 27.7% for Mexican American women (153). In the HCHS/SOL, the age-adjusted prevalence of hypertension was 25.5% (154).

In CARMELA, hypertension prevalence ranged from 7.2% (men) and 10.1% (women) in Ecuador to 37.7% (men) and 21.7% (women) in Argentina (155). LASO reported a mean prevalence of 20.2% (133). CRONICAS reported a prevalence of 23.9% for individuals without diabetes, and 50.9% for individuals with diabetes (139). ELSA-Brasil reported a mean prevalence of 25.7% in women and 34.5% in men (149). In CESCAS I hypertension prevalence ranged from 36.9% in Temuco, Chile to 45.3% in Bariloche, Argentina, and a mean of 40.8% in Chile, Argentina, and Uruguay (136).

#### Dyslipidemia

In the 2011–2012 NHANES cycle, prevalence of high total cholesterol was 13.5% in NHWs and 14.2% in Hispanics/Latinos, and the prevalence of low high-density lipoprotein cholesterol (HDL-C) was 17.1% in NHWs and 21.8% in Hispanics/Latinos (156). In the HCHS/SOL, the prevalence of elevated total cholesterol was 13.9% in women and 14.3% in men, elevated low-density lipoprotein cholesterol (LDL-C) was 34% in women and 38.2% in men, triglycerides (TGs) > 200 mg/dL was 10.2% in women and 19.8% in men, and low HDL-C was 47.9% in women and 34.3% in men (157).

In CARMELA, the prevalence of hypercholesterolemia ranged from 5.7% in Barquisimeto, Venezuela to 20.2% in Quito, Ecuador (128). CESCAS I reported an overall prevalence of hypercholesterolemia of 24.4% (which ranged from 20.8% in Bariloche, Argentina to 31.0% in Barros Blancos, Uruguay), high LDL-C of 23.1%, low HDL-C of 34.1%, and hypertriglyceridemia of 22.1% (136). LASO reported an overall prevalence of 8.9% of elevated total cholesterol, 8.5% of high LDL-C, 53.3% of low HDL-C, and 26.5% of high TGs (133). ELSA-Brasil reported a mean prevalence of hypercholesterolemia of 68.2% in women and 65.5% in men (149). Although the prevalence of lipid and lipoprotein abnormalities varies by region and study, it seems like low HDL-C is highly prevalent across Hispanic/Latino populations in the US and Latin America.

#### Tobacco Use

The prevalence of smoking among US adults is 16.7% for men and 13.6% for women, with distinct racial/ethnic differences (158). The prevalence among NHWs is 20.2%, and 10.1% for Hispanics/Latinos, nationwide. However, the HCHS/SOL demonstrated a higher overall prevalence of daily smoking (16.9% in men and 10.7% in women) and significant differences among heritage groups with higher prevalence of daily smokers in Puerto Rican men (27.0%), Cuban men (26.2%) and Puerto Rican women (24.2%), and lower smoking prevalence in Dominican men (8.8%) and Mexican women (4.4%) (159).

In Latin America, the CARMELA reported a prevalence of current smoking that ranged from 21.8% in Barquisimeto, Venezuela to 45.4% Santiago, Chile (128). LASO reported a mean prevalence of 25.8% (133), and CESCAS I reported a mean prevalence of 29.7% in Argentina, Chile, and Uruguay (136). ELSA-Brasil reported a mean prevalence of current smoking of 15.5% in women and 16.7% in men (149). In contrast, CRONICAS reported a prevalence of daily smoking of 3.3% among individuals without diabetes, and 2.7% among those with diabetes in Perú (139).

#### Behavioral Risk Factors

Cardiometabolic factors are influenced by modifiable behavioral risk factors, namely physical activity (PA), and dietary intake. The 2010 Racial and Ethnic Approaches to Community Health Risk Factor Survey estimated approximately one-third of Hispanic/Latino men and women met PA recommendations (160). Moreover, using objective measures, the HCHS/SOL reported that Hispanic/Latino adults spent 11.9 h/day, or 74% of their monitored time, in sedentary behaviors, with less time for Mexicans and more for Dominicans (161). CESCAS I reported a prevalence of physical inactivity of 35.2% (136); ELSA-Brasil reported a mean prevalence of physical inactivity of 79.8% in women and 70.9% in men (149).

In the HCHS/SOL, differences in total energy, macronutrient, and nutrient-dense intakes were observed among the different heritage groups (162), with higher total energy and macronutrient intakes by Cubans, higher fiber intake by Mexicans, lower fiber intake by Puerto Ricans and lowest total energy intake by Dominicans. Evaluations of the dietary intake and quality based on the 2010 Alternate Health Eating Index and the Dietary Approaches to Stop Hypertension (DASH) revealed overall low scores for intakes of whole grains, and fruit and vegetables with lower dietary quality for Puerto Ricans and higher quality for Mexicans (163, 164).

Dietary patterns in Latin America have been gradually shifting toward increased consumption of sugars and meat, and decreased consumption of fruit and cereals across the continent (165, 166). In CESCAS I, 85.5% of participants reported low intake of fruits and vegetables (136). CRONICAS reported that 3.8% of individuals without diabetes and 9.5% with diabetes consumed 5 or more servings of fruits/vegetables per day (139). ELSA-Brasil reported low health dietary scores (1.23–1.38 out of 5) (149).

## Etiology of Type 2 DM in Hispanics/Latinos

Multiple factors contribute to type 2 DM risk including: genetic variations, demographic characteristics, and behavior- and lifestyle-related risk factors (167). Among the pathophysiologic defects in type 2 DM, insulin resistance along with β-cell failure are considered major defects (168). Alterations in the adipose tissue (endocrine organ), the gut incretin-system (enteroinsular axis), renal reabsorption of glucose, and brain insulin response also play important roles in the development of glucose intolerance and type 2 DM (169). In addition, alterations in the crosstalk between immune and metabolic pathways are closely linked to obesity and diabetes (170). Inflammation is also recognized as a common pathway for the major complications of atherosclerosis, stroke, and ischemic heart disease, commonly observed in persons with diabetes (171). Some of these aspects have been studied in Hispanics/Latinos.

### Obesity and Insulin Resistance

As reported in other ethnic groups, type 2 DM is associated with obesity in Hispanics/Latinos (172). However, obesity does not explain the excess diabetes prevalence among Hispanics/Latinos compared to NHWs (172). For instance, 29.3% of participants of HCHS/SOL with prediabetes had normal weight (95). On the other hand, duration of obesity or earlier age of onset has been associated with increased risk of developing type 2 DM (173–175).

In addition to overweight or obesity, the distribution of the adipose tissue has been associated with increased risk for type 2 DM and CVD. Non-alcoholic hepatic steatosis, for example, has been described in some Hispanic/Latino groups, and has been linked to increased insulin resistance and other cardiometabolic abnormalities (176, 177).

Data from the SAHS showed that insulin resistance measured by several insulin resistance surrogates accounts for a significant proportion of the excess type 2 DM risk in Mexican-Americans compared to NHWs (178). This study also showed evidence of higher all-cause and CV mortality among Hispanics/Latinos born in the US (179).

### Adipokines, Hypercoagulability, Inflammation, and Endothelial Dysfunction Biomarkers

Several adipokines and inflammation and hypercoagulability mediators, such as leptin, intercellular adhesion molecule-1, tissue plasminogen activator, inhibitor of plasminogen activator-1, high-sensitivity C-reactive protein (CRP), macrophage chemoattractant protein 1 (MCP-1), tumor necrosis factor (TNF)-α, and interleukin-6 (IL-6) have been found to be elevated in young Hispanics/Latinos at high risk of developing type 2 DM (180–189) and individuals from different racial/ethnic groups—including Hispanics/Latinos—with type 2 DM (189–194). Elevated IL-6, leptin, CRP, and TNF-α have been observed in Mexican Americans with type 2 DM, and the elevation IL-6 and leptin in particular was linearly associated with increasing glycemia (194).

For example, in a prospective study of Hispanic/Latino adolescents with obesity, higher baseline MCP-1 or IL-6 levels at prepubertal age were associated with a 16 and 21% greater decline in insulin sensitivity during puberty (195). However, this relationship between proinflammatory/endothelial dysfunction markers and insulin sensitivity has been shown to vary with adipose tissue distribution, and may differ across racial/ethnic groups (188, 192). Adiponectin, a collagen-like protein secreted from adipose tissue has been inversely correlated with both insulin sensitivity and adiposity (196–198), and increased risk of developing type 2 DM in Hispanic/Latino adults (199). Adiponectin inversely correlates with CV risk factors in older adults (200), and appears to predict progression to glycemic failure in adolescents with type 2 DM from different ethnic groups (200, 201). A cautionary note: Most of these studies have been small, and the representation of Hispanic/Latino heritage groups has been very limited.

### Other Factors

*Maternal factors* such as fasting indexes of total triiodothyronine, insulin, leptin, and ghrelin have been associated with excessive weight gain in Hispanic/Latino children (202–204). Women who experience GDM are at high risk of developing GDM in subsequent pregnancies (205, 206) and type 2 DM (205). In addition, children born to women with GDM are at increased risk of obesity and glycometabolic disease later in life (204–214). Increased proinflammatory markers have also been identified in women with GDM (215). Research studies involving Hispanic/Latino women in the US or in Latin America have described increased proinflammatory markers (e.g., proinflammatory cytokines and/or natural killer cells) at the placental level (216–218), and at both the placental and fetal level (219) associated with maternal hyperglycemia during pregnancy. In Mexican women, prepregnancy overweight and obesity have been associated with increased oxidative stress in the newborn (220). In Chile, in women with overweight or obesity and GDM, maternal elevated TGs were associated with increased infant’s birth weight (BW), despite good maternal glucose control (221). Maternal hyperglycemia, whether due to preexisting diabetes or GDM, has been associated with increased oxidative stress and maternal DNA damage (222, 223), and both placental and umbilical cord DNA damage (222, 223). However, the number of patients in these studies has been small, and the interaction of obesity or other proinflammatory factors cannot be separated from hyperglycemia. Whether these changes could be prevented or reversed is unknown.

Low BW, like maternal GDM, has been inversely associated with glucose intolerance and insulin sensitivity during childhood and increased risk for CVD later in life. This susceptibility to adult chronic diseases may be a response to exposures *in utero*—known as fetal programming—or early malnutrition in extra uterine life (224, 225). Several studies in Latin America have documented the inverse association between BW and insulin sensitivity later in life (226), malnutrition during the first year of life and insulin sensitivity, glucose tolerance and metabolic syndrome later in life (227–229); and being exclusively or predominantly breastfed > 12 months and lower adiposity and serum cholesterol during childhood (230).

*Chronic malnutrition* is associated with alterations in glucose metabolism and insulin sensitivity (231). Decades ago, a malnutrition-modulated diabetes or tropical diabetes was described in developing countries (232, 233). Some investigators hypothesized that consumption of cassava (manioc or tapioca) could lead to diabetes in malnourished subjects, due to cyanogenic glycosides leading to chronic pancreatitis; however, the theories remained largely speculative (232). Others postulated that chronic protein energy malnutrition would cause persistent insulin deficiency and glucose intolerance without ketosis, among other clinical features (234–236).

A link between plasma *branched-chain amino acids* (BCAAs) and insulin clearance has been observed in recent years. Overweight persons exposed to a diet high in saturated fat have been found to have a significant inverse correlation between plasma BCAAs and insulin clearance. This association has also been observed in Hispanics/Latinos (237).

Atypical forms of diabetes such as *ketosis prone diabetes* (KPD) have also been described in Hispanics/Latinos. KPD affects 20–50% of African-American and Hispanic/Latino patients with new diagnoses of diabetic ketoacidosis (238). Hispanics/Latinos with KPD also appear to be disproportionally affected by KPD characterized by an absence of autoantibodies and presence of β-cell functional reserve (239, 240).

Sleep disruptions characteristic of *obstructive sleep apnea (OSA)* and that alter sleep duration and timing may promote behavioral, metabolic, and/or hormonal changes associated with changes in weight (241–244). OSA is independently associated with insulin resistance (245) and predicts subsequent risk for type 2 DM (246). In the HCHS/SOL, both quantity and quality of sleep were associated with higher odds of having type 2 DM (247, 248) and impaired glucose tolerance (IGT) (247) with the greatest odds among those with short sleep duration and insomnia (249). Three loci have been found to be significantly associated with OSA traits in a large genome-wide association study of 12,558 Hispanics/Latinos (250). The association of fasting blood glucose and OSA was weaker among Hispanics/Latinos in MESA compared to African Americans (AA) and NHWs (251).

In recent years, *Alzheimer*’*s disease* (AD) has been considered as a metabolic disease mediated by alterations in brain insulin responsiveness, glucose utilization, and energy metabolism that can lead to increased oxidative stress, inflammation, and worsening insulin resistance (252). The association between type 2 DM and AD has been called type 3 diabetes (253). Mexican American elders with diabetes are at almost twofold increased risk of dementia than those without diabetes (254). Also, longer duration of diabetes has been associated with a faster rate of cognitive decline in this age group (255). Further research is needed to examine the interaction of type 2 DM and AD in this vulnerable group, and among other Hispanic/Latino groups.

Other emerging risk factors for type 2 DM and CVD include the gut microbiome (256–258), gallbladder disease (259), environmental exposures (260–262), and the impact of sugar-sweetened beverages intake on satiety (263). Research on these emerging factors in Hispanics/Latinos has been limited.

### Genetics and Type 2 DM in Hispanics/Latinos

The polygenic nature of type 2 DM is well established and more than 100 loci for type 2 DM and glycemic traits have been identified through genome-wide association studies (GWAS) of common and rare variation in populations of diverse ancestral origins (264).

Genetic variations in the gene encoding for transcription factor 7-like 2 (TCF7L2) have been associated with type 2 DM in different populations (265). The T allele of single nucleotide polymorphism (SNP) rs7903146 of TCF7L2 strongly predicts the development of type 2 DM (265, 266). TCF7L2 has been associated with GDM and type 2 DM in Hispanics/Latinos of Mexican descent (267, 268), and polymorphisms of this gene have been associated with reduced acute insulin response in this heritage group (269). This gene has also been associated with diabetes in the HCHS/SOL (270). TCF7L2 polymorphism rs7903146 has also been associated with coronary artery disease (271, 272) and stroke (273) among patients with long standing history of type 2 DM.

A haplotype containing four missense SNPs, all in SLC16A11, conferring 20% increased risk for type 2 DM was identified through GWAS in Mexican and other Latin American samples in the SIGMA type 2 DM Consortium (274). A newly identified African ancestry-specific allele at KCNQ1 was associated diabetes in HCHS/SOL (270), however, the mechanisms leading to type 2 DM due to alterations in this allele are less well understood (275).

Whole-exome sequencing in 3756 individuals of Mexican and Mexican American ancestry identified a rare variant (p.E508K) in HNF1A that had significant association (fivefold increase) with type 2 DM (276). HNF1A is the gene responsible for MODY3, a monogenic, early-onset form of type 2 DM, however carriers of p.E508K did not show early-onset of type 2 DM and were indistinguishable from the wider type 2 DM population, thus not fulfilling the classical diagnostic criteria for MODY3 (276).

The Genetics Underlying Diabetes in Hispanics/Latinos (GUARDIAN) Consortium (277) conducted a GWAS in multiple Mexican-ancestry cohorts with highly detailed glucose homeostasis measures. Nonparametric meta-analysis of the Discovery and Translation cohorts identified a significant relationship with type 2 DM phenotype at 6p24 (SLC35B3/TFAP2A) in association with glucose effectiveness, 11p15 (KCNQ1) with disposition index, and 6p22 (CDKAL1) and 11q14 (MTNR1B) with acute insulin response (277).

Genetic studies of GDM in Latin American women have yielded varied findings. In Brazilian women, GDM has been associated with the glucokinase gene (278), the MTNR1B gene polymorphism rs10830963 (279), LGALSI polymorphism (280) and IRS-1 (281). In Mexican women, GDM has been associated with polymorphisms of the HNF4A gene (282), the TNF-α gene promoter (283), the MTNR1B gene (284), the CENTD2 gene (284), the KCNQ1 haplotype (284), and the TCF7L2 gene (284, 285). However, polymorphisms of the fat mass and obesity-associated (FTO) and the TCF7L2 genes have not been associated with GDM in Brazilian women (286), and SLC16A11 locus was not associated with GDM in Mexican women (284).

Among Mexican American subjects recruited in the San Antonio Family Heart Study, DNA methylation levels at five CpG sites, mapping to three well-characterized genes (TXNIP, ABCG1, and SAMD12) independently explained 7.8% of the heritability of type 2 DM (287). In the same population, individuals with the hypertriglyceridemic waist phenotype [waist circumference ≥95 cm in men and ≥80 cm in women] combined with high serum TG concentration (≥2.0 mmol/L in men and ≥1.5 mmol/L in women) were found to have epigenetic changes (DNA methylation) in genes involved in β oxidation of long-chain fatty acids (CPT1A) and triglyceride storage (ABCG1) (288).

In summary, these findings have shown the broad complexity of the pathophysiology of metabolic disease and research focused on Hispanics/Latinos. Broader attention to different heritages should be considered in future studies to better stratify subjects at risk for type 2 DM and develop intervention strategies according to different genotypic and phenotypic traits.

## Interventions to Prevent Type 2 DM among Hispanics/Latinos

### Lifestyle Interventions to Prevent Type 2 DM in Hispanic/Latino Women with History of GDM

The increasing prevalence of obesity and metabolic disease in young Hispanics/Latinos (and other racial/ethnic groups) has raised thought-provoking questions about the role of maternal health and the intrauterine environment on the child’s future risk for type 2 DM and obesity. As previously described, women who experience GDM are at high risk of developing GDM in subsequent pregnancies and type 2 DM, and their children are at increased risk of metabolic disease.

The quality of dietary fat intake (289) and PA (290–293) during mid-pregnancy have been as proposed factors that could influence the risk of GDM in Latin American women. However, clinical studies evaluating the effect of exercise training on pregnancy outcomes have yielded inconsistent or even conflictive results (292, 294). In a systematic review by Perales et al., the authors concluded that the combination of aerobic and resistance training during pregnancy seemed to produce the most favorable effect on different maternal health parameters, but that this combination (or either type of exercise separately) yielded an overall weak effect on reducing GDM (294). Previous history of GDM, preexisting overweight or obesity, gestational weight gain, intensity, frequency, duration, and timing of exercise, and adherence to the PA regime may be factors impacting program efficacy (292, 294).

Research focused on preventing GDM or type 2 DM in women with a history of GDM—especially Hispanics/Latinas—has been scarce. Some studies have evaluated PA and weight gain/retention during pregnancy or postpartum (295–297). In the Behaviors Affecting Baby and You Study (*n* = 110 women at risk of GDM, 60% Hispanic/Latina) participants were randomized to either a 12-week tailored PA intervention or usual wellness care (295). Women on the tailored exercise intervention had smaller decrease in postpartum PA than the control group (−1.0 MET-hours/week versus −10.0 MET-hours/week). In the Diet Exercise and Breastfeeding Intervention Study (*n* = 116, 35.5% Hispanic/Latina), pregnant women with GDM were randomized to either a Diabetes Prevention Program (DPP)-modeled intervention that started during pregnancy and continued postpartum, or usual care to determine feasibility and changes in metabolic parameters (296). In a subsample of 72 participants, women who had lost > 2 kg experienced lower increases in fasting glucose, 2h-glucose and homeostasis model assessment of insulin resistance (HOMA-IR) at 12 months postpartum. Hispanic/Latino women were more likely to maintain or gain weight postpartum than women from other racial/ethnic groups (296).

In the Gestational Diabetes’ Effects on Moms Study (*n* = 2,280 women with GDM, 22.2% Hispanic/Latina), a DPP-modeled intervention between 6 weeks and 6 months postpartum was compared to a usual care (mailed recommendations only) (297). The intervention arm had 28% higher odds of meeting postpartum weight goals than usual care. A greater proportion of women on the intervention arm had less weight retention at 6 weeks (25.5 vs. 22.4%) and 6 months postpartum (30.6 vs. 23.9%, mean weight lost = 1.89 vs. 0.94 kg) compared to the women in usual care. However, the difference was no longer significant at 12 months (33.0 vs. 28.0%, mean weight lost = 1.19 vs. 0.50 kg). Also, at 6 months postpartum, the intervention arm had a greater increase in vigorous-intensity PA (mean 15.4 min/week) than usual care.

In the Parish Nurse Intervention Program (298), 100 Mexican American women with GDM were randomized to an interview-based intervention assessing health-promoting behaviors and lifestyle or to usual care. At postpartum, intervention participants reported higher scores on health-promoting behaviors than women receiving usual care. While promising, there were no significant differences in blood glucose, HbA1c, macrosomia, and other biometric parameters. In the Dulce Mothers Project, 84 Hispanic/Latino women with previous GDM were randomized to a shortened 8-week DPP-modeled intervention, or usual care (299). After 6 months, intervention participants reported a significantly higher PA and lower fat intake, but there were no changes in body weight or BMI. Although changes in HbA1c% (5.73–5.82%), total cholesterol (180–169.9 mg/dL), LDL-C (107.8–100.4 mg/dL), and TGs (124–110.3 mg/dL) were statistically significant, they were not clinically significant. In the Estudio Vida (300), 68 pregnant Hispanic/Latino women of unspecified ancestry were randomized to a 6-month culturally and linguistically modified lifestyle intervention (monthly in-person behavioral counseling, and five telephone-delivered booster sessions, plus follow-up 6 weeks postpartum) or to usual care, to compare PA, gestational weight gain, infant BW, and biomarkers of insulin resistance postpartum. Although the intervention was shown to be feasible, no changes were observed in any of the outcomes (300).

Some of the studies described above did not report the Hispanic/Latino heritage group of the participants and/or assess cardiometabolic markers. In addition, it is unknown whether the follow-up of participants continued beyond the intervention period, and whether the risk for future GDM or type 2 DM was reduced. Other interventions focused on Hispanic/Latino women with history of or at risk of GDM and involving lifestyle intervention have been proposed (301, 302) have been proposed or initiated. However, results have not yet been published.

### Lifestyle Interventions to Prevent Type 2 DM in Hispanic/Latino Youth in the US and Latin America

Little information is available of type 2 DM prevention efforts focusing on the youth (303). To date, no published investigations are available to address type 2 DM prevention in Hispanic/Latino youth with prediabetes. Although programs designed to prevent childhood and youth obesity have been extensively reviewed, these seldom assess blood pressure, lipids and lipoproteins and other metabolic markers, or adiposity (304–311). The effectiveness trials for prevention of type 2 DM among youth are further challenged by a lack of consensus on parameters for disease risk indicators (adiposity, CV risk markers, resting energy expenditure), especially during puberty, and its associated changes in body composition, insulin sensitivity and or insulin secretion (312–317).

#### Weight Loss and Obesity Prevention Trials

Weight loss interventions focusing on children with overweight or obese have resulted in improved metabolic outcomes such as insulin sensitivity, lipids and adiposity measures (318–324). Most of these trials involved medium to high-intensity interventions focusing on physical activity (PA) and dietary intake (309, 311, 325). Interventions delivered in schools with home involvement (PA-only interventions), or with home and community components (diet and PA interventions) have produced the strongest evidence of the metabolic benefits of weight loss and, consequently, childhood obesity prevention in the US and Latin America (310, 325, 326).

While promising, there are few weight gain prevention interventions specifically focusing on Hispanics/Latinos in the US and Latin America (304, 308, 327). Reviews of childhood obesity interventions in Latin America and the Caribbean report encouraging trends in childhood obesity prevention and treatment programs in schools (328), healthcare (329), and settings such as home, summer camps and family clinics (330). However, these studies were mostly carried out in Mexico and Brazil, only three were randomized clinical trials (RCTs), and most did not include appropriate pediatric measures for BMI or participants’ ages (327). In the US, evaluations of community-based interventions targeting multiple predictors of childhood obesity are underway *via* the CDC-funded Childhood Obesity Research Demonstration Projects (CORD) (331–334). Two of the sites will focus specifically on Hispanic/Latino populations.

##### Dietary Considerations

Although there is no prescribed diet for diabetes prevention among youth, reductions of saturated fats and sugars and increases in fruits, vegetables, and fiber are expected to prevent overweight among children (335, 336) and therefore are also used in weight loss intervention trials. However, some argue that diets for type 2 DM prevention among youth should instead focus on low-glycemic foods (337). Small trials that test the safety and efficacy of intense diet modifications suggest that low-glycemic food choices decrease waist circumference, BMI z-score, and insulin resistance—assessed through the HOMA-IR—compared to higher glycemic food choices (323, 325, 337–341). Some authors argue that these diets lead to greater decreases in adipose tissue compared to low-energy and low-fat diets (338, 342). In terms of metabolic effects of these diets specifically among Hispanics/Latinos, evidence is still emerging (325).

##### Physical Activity Considerations

Intervention trials involving exercise only (without diet restrictions) among youth and adults have shown effects on metabolic outcomes and cardiorespiratory fitness without affecting weight or fat mass (343–349). Data specific to Hispanics/Latinos are limited, but in Mexico, Macías-Cervantes et al. observed a significant decrease in insulin and HOMA-IR after a randomized trial of a 12-week exercise training intervention in a RCT of 76 children, aged 6–9 years, with no significant changes in BMI or diet between control and intervention groups (347). Of note, the ADA recommends that children engage in 60 minutes of exercise per day most days, and that they limit time in front of a screen to less than 2 hours a day as a type 2 DM prevention strategy in children (336).

#### Preventing Prediabetes in School-Aged Children

DPPs in school settings have been somewhat successful at increasing PA (and sometimes improving diet) *via* education and hands-on activities. However, their effects on BMI, adiposity measures, and lipids have been inconsistent (305, 350–353). Results are difficult to compare due to the range of both the cardiometabolic risk indicators assessed and the age of the children included in the studies.

Lifestyle intervention trials have been conducted in schools with large (>50%) Hispanic/Latino populations to achieve change in metabolic and anthropometric outcomes among normal-weight students. These interventions have often involved PA and healthful nutrition education components and activities that are incorporated into the schools’ curricula (regular and PA classes) and cafeteria. After 4–7 month interventions involving elementary- and middle-school children, several studies reported significant improvements in behavioral or metabolic outcomes, without an effect on anthropometrics (354–356). Two additional studies with similar school-based programs reported improvements in metabolic and anthropometric parameters in the US and Brazil (350, 357), yet, not all studies have yielded significant results. For example, the HEALTHY study, a larger school RCT in seven centers in the US in which children in middle school (sixth to eighth grade, 54.2% Hispanics/Latinos) were followed, did not observe significant decreases in overweight/obesity, lipid, hypertension, or other metabolic markers among Hispanics/Latinos (358, 359).

Studies that focus on older Hispanic/Latino youth (aged 14–17 years) are less consistent in their findings. A small RCT in Brazil (360) reported that a high-intensity exercise intervention for 43 adolescents with obesity did not improve BMI, waist circumference, blood glucose, physical fitness, HDL-C, or lipids. Other studies in older Hispanic/Latino teenagers (14–17 years) with obesity in the US or Mexico used similar dietary intake-targeted interventions for an average 4-month period. Although they reported significant changes in some anthropometric measures, physical inactivity, diet, and cardiometabolic risk factors, they were insufficiently powered and did not include controls (361–363).

### Lifestyle Interventions to Prevent Type 2 DM in Hispanic/Latino Adults in the US and Latin America

The evidence for successful type 2 DM prevention interventions among adults is growing. However, the increasing prevalence and incidence of type 2 DM among Hispanic/Latino adults suggest that preventive programs have not caught up with the needs of this population.

#### The DPP

The DPP is a seminal type 2 DM prevention trial (364). It recruited an ethnically diverse (*N* = 3,234, 16% Hispanics/Latinos) cohort and established a multisite program that demonstrated that a lifestyle intervention targeting 7% body weight reduction and increase in PA (goal = 150 minutes per week) effectively reduced by 58% (compared to placebo) the incidence of type 2 DM among persons at high risk, and compared to 31% reduction with metformin (364, 365). The reduction of diabetes incidence was similar among race/ethnic groups, including Hispanics/Latinos (364). The percent of weight loss achieved by Hispanics/Latinos in the lifestyle intervention arm (women 7.3%, men 7.7%) was close to that of NHWs (women 7.9%, men 8.5%) (366).

#### Lifestyle Interventions Modeled after the DPP in Hispanics/Latinos in the US

The success of the DPP has sparked numerous attempts to modify this individual-based intervention for delivery in various community settings serving culturally diverse groups (367–379). Many of these translations appear to be effective for weight loss and improvement in cardiometabolic parameters (368, 379), although questions remain as to appropriate dosage that makes interventions both accessible and cost-effective, as well as the way in which these interventions have been culturally adapted to different populations.

A recent review identified only five RCTs designed to evaluate lifestyle interventions aimed to reduce risk of type 2 DM in Hispanics/Latinos (measured by reduction of weight or HbA1c) (380), and only two were deemed to have a strong quality rating. These two studies were conducted in women only (370) or mostly (>70%) (368), and only one of them reported a significant reduction in HbA1c (368).

Most studies designed for type 2 DM prevention among Hispanics/Latinos culturally tailor their programs to the population of interest (367–369, 371, 381). A recent review of culturally tailored type 2 DM prevention interventions in US Hispanic/Latino adults described the varied approaches to cultural tailoring, which ranged from use of bilingual materials to community input on program content (380). As is the case in virtually all culturally adapted studies, none assessed the relative importance of various cultural adaptation elements, did not compare culturally adapted interventions and non-adapted approaches (380), and did not specify participants’ ancestry or country of origin. While budgetary limitations often present barriers to these analyses, it would be important to ascertain the usefulness of elements of cultural adaptation, as well as to determine the variability of health-related behaviors by cultural origin (84, 163, 382–384). There is a critical need for culturally adapted interventions to provide more detailed information on the methodology followed in the cultural adaptations, beyond the translation of materials, as well as to address the linguistic needs of many Latin American immigrants from indigenous communities, for whom Spanish is not a first language.

In Latin America, there seem to be no published studies modeled after the DPP, but several RCTs have evaluated the effect of lifestyle interventions on weight and metabolic outcomes among adults in Mexico and Brazil (384). The RCTs varied in size (*N* = 51–241), intervention dosage (weekly to monthly), duration (6–12 months), and specific intervention components. The dietary interventions included the DASH diet (385), low-glycemic diets (386), or diets recommending olive oil, fruits, and vegetables (387). The studies reported significant changes in body weight loss and/or metabolic markers, including fasting blood glucose, HbA1c, and lipids (386–390).

### Pharmacological Interventions to Prevent Type 2 DM in Hispanic/Latino Adults in the US and Latin America

A few pharmacological interventions focused on preventing or delaying type 2 DM in Hispanics/Latinos have been published (391–395). The PPAR γ agonists troglitazone (391) and pioglitazone (392) have demonstrated long-term preservation of β-cell function in Hispanic/Latino women with a history of GDM. Troglitazone significantly reduced the annual incidence rate of type 2 DM (5.4 versus 12.1% for placebo) over a median 30-month follow-up (391), and pioglitazone demonstrated a significant decrease over 1-year period (392).

Boyko et al. reported a significant and greater reduction in diabetes incidence (hazard ratio = 0.18 over approximately 5 years, *P* = 0.0242) in Latinos taking rosiglitazone, another PPAR γ agonist, and South Asians showing the lowest reduction (393). De Fronzo et al. reported a similar effect of pioglitazone at reducing diabetes incidence (hazard ratio = 0.28 over 2.4 years, *P* < 0.0001) among NHWs, AAs and Hispanics/Latinos compared to placebo (394).

O’Brien et al. (395) compared a 12-month intensive lifestyle intervention (DPP-modeled) led by *promotoras* to metformin, and to usual care in a group of 85 Hispanic/Latino women (aged ≥ 20 years) with prediabetes (IFG and/or HbA1c 5.7–6.4%). The investigators observed a significant reduction in weight (−4.0 kg or 5.0%, *P* < 0.001) as well as in BMI in women on the lifestyle intervention compared to the other groups. However, no significant changes in blood pressure, biochemical parameters, or HOMA-IR were observed. Of note, the mean adherence to metformin was 66.4%.

#### Pharmacological Interventions for Cardiometabolic Risk Factors in Hispanic/Latino Adults in the US and Latin America

Pharmacological interventions specifically designed to improve cardiometabolic risk factors in Hispanics/Latinos at high risk of type 2 DM are limited. Despite this limitation, we would like to highlight some key findings.

Four RCTs (396–400) designed to compare the effectiveness of combinations of angiotensin-converting enzyme (ACE) inhibitors or angiotensin II receptor blockers (ARBs) on blood pressure reduction and/or prevention of CV events with other antihypertensive medications reported that Hispanics/Latinos demonstrated good response to the therapies and maintenance of blood pressure control, similar or even superior than that observed in NHWs (396–399). In one of the trials, the addition of trandolapril to the treatment was associated with a lower incidence of diabetes and CV events among Hispanics/Latinos than in non-Hispanic participants (398). This observation is consistent with previous clinical trials (in which Hispanics/Latinos had not been included) that reported a lower incidence of type 2 DM associated with ACE inhibitors and ARBs (401, 402). Also, a secondary data analysis on one of the trials revealed that a significantly larger percent (75%) of Hispanic/Latino women (*n* = 5,017) reached blood pressure goals compared to 68% of NHW women (*n* = 4,710), and that Hispanic/Latino women experienced fewer CV events (5.7 versus 12.3%) (403). In addition, Punzi et al. reported a significant reduction in blood pressure in Hispanics/Latinos with Stage I-II hypertension associated with nebivolol (β blocker) (404). Blood pressure control was achieved by 32–71% of participants depending on the dose. Some of these studies reported the Hispanic/Latino heritage group of the participants or the Latin American countries where the studies were conducted. Other studies did not report nationalities or heritage group.

The results of the studies described above suggest Hispanics/Latinos experienced similar or better response to antihypertensive therapy than patients from other demographic groups (405). In contrast, Yi et al. did not observe an increased blood pressure control with self-blood pressure monitoring in a low-income urban setting (406). Thus, the good response to hypertensive medications observed among Hispanics/Latinos in clinical trials may be both physiological and dependent on a controlled research setting. Future research may address underlying self-efficacy and contextual barriers that impeded the success of the self-monitoring and blood pressure control outside of clinical trials.

Various clinical trials evaluating the efficacy of lipid-lowering agents have reported significant reductions of total cholesterol and LDL-C, or CRP in US Hispanics/Latinos or Latin Americans (407–410), and comparable to reductions experienced by NHWs (407, 408). These studies have also reported good medication tolerance.

### Policies and Priorities and Diabetes Prevention Research in Latin America

Countries throughout Latin America and the Caribbean are starting to raise awareness about chronic disease, including type 2 DM treatment and prevention. For example, the Latin American Association for Diabetes published guidelines about the treatment and management of type 2 DM across medical associations in 17 countries (411). During the 2016 Pan American Health Organization (PAHO) symposium on Diabetes in the Americas, various countries described the epidemiology of diabetes in terms of incidence, prevalence, mortality, and risk factors. The review discussed policies or public awareness programs in place or designed to raise awareness of diabetes management focused mostly on nutrition education and physical activities (412).

Increased activities about prevention and treatment of type 2 DM, such as those described during the PAHO Diabetes in the Americas symposium, can be leveraged to increase our understanding of whether strategies designed to target type 2 DM risk factors (i.e., prediabetes, hypertension, and obesity) can actually lead to lower disease risk. As of May 2015, Chile and Mexico had implemented national-level taxes on sodas, and evaluations for these programs are underway (413). Various Latin American countries have set strategies, which have not yet been evaluated, to increase awareness about the harmful effects of transfatty acids (414) and of high salt intake (415–419). Other population-level initiatives include the creation of guidelines for healthful eating and PA; the creation of *Ciclovía-Recreovía* in Colombia (420), additional training of health care professionals to improve diabetes care, and diabetes screening initiatives in Chile, Argentina, and Honduras, nutrition labeling in Ecuador, and the ban of unhealthful foods in schools in Costa Rica, Perú and Guatemala (418). These new strategies present a unique opportunity to evaluate the process of implementation, the role of stakeholders in their design and implementation, and their eventual impact on anthropometric, lipids, and metabolic indicators or diabetes prevalence and treatment.

## Preventing Type 2 DM in Hispanics/Latinos—A Holistic Framework

The epidemiology, etiology, and interventions studies selected for this review provide important insights on the magnitude of the prevalence, the complexity of potential mechanisms of disease, and the approaches toward preventing type 2 DM in Hispanics/Latinos. Based on these insights, it is evident that the successful prevention of type 2 DM in Hispanic/Latino populations in the US and Latin America would need a fundamentally radical transformation. In the era of personalized medicine, this transformation would require a holistic mission and a multidisciplinary approach.

**Figure 2:**
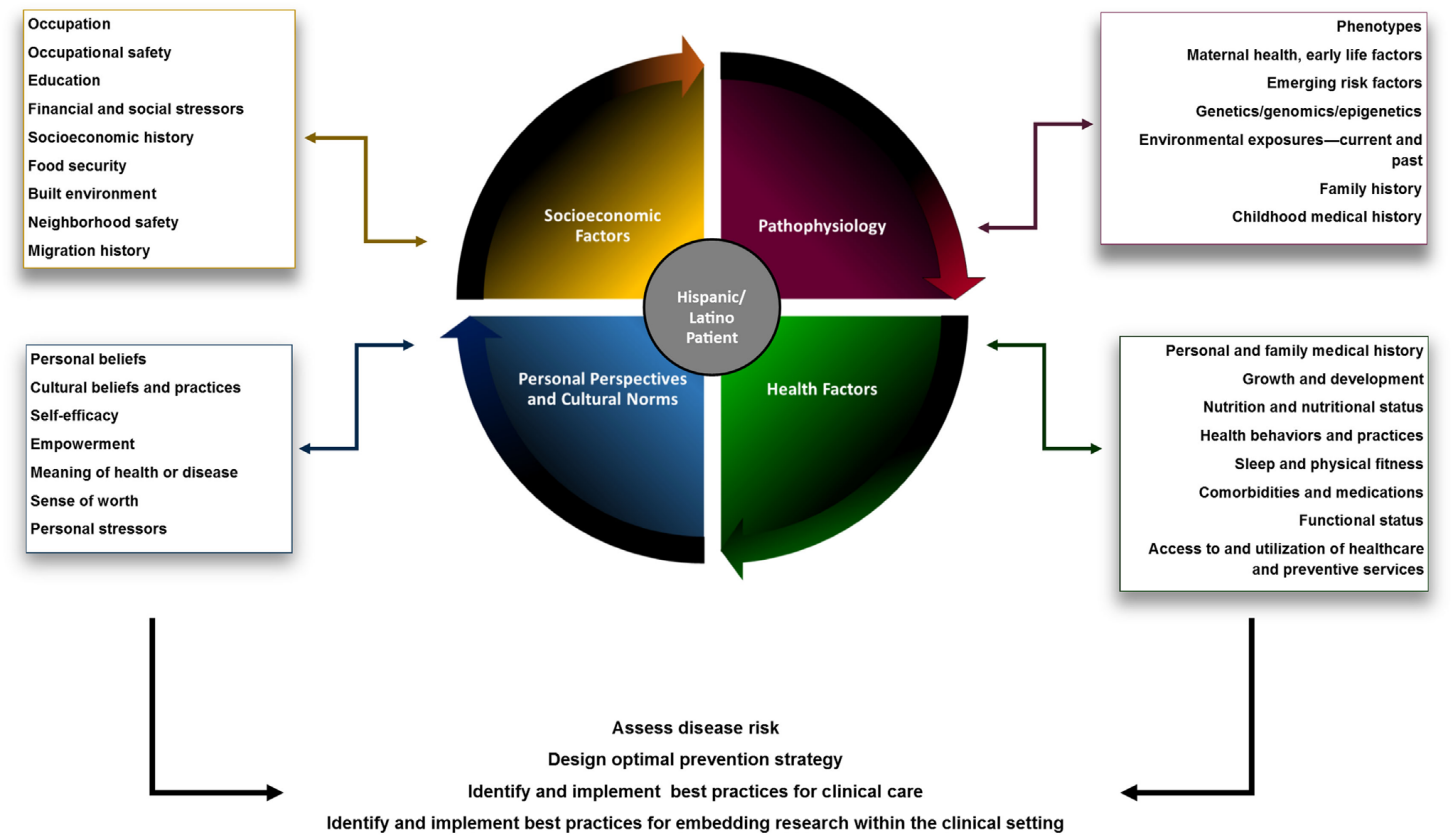
Framework of opportunities to enhance research and clinical care oriented toward preventing type 2 diabetes in Hispanics/Latinos in the US and Latin America.

This holistic framework integrates key domains that interact with each other (Figure 2). Within each domain we have identified areas that represent opportunities for enhancing the prevention of type 2 DM in Hispanic/Latino populations—whether as a part of a research program or clinical care intervention. The Hispanic/Latino individual is at the center, and representing the most important element of the framework.

Our understanding of the burden of diabetes (type 1 and type 2) in Hispanic/Latino populations remains insufficient and should be prioritized. The development of effective type 2 DM preventive efforts requires continued surveillance of the burden of disease and its risk factors using disaggregated data on Hispanic/Latino subgroups. Adequate representation of diverse populations would enhance the identification of different phenotypes, and potential disease mediators and interactions. More detailed epidemiologic data at the country level in Latin America are urgently needed.

Consistency and standardization of methodology to assess the prevalence of diabetes in Hispanic/Latino populations would more accurately estimate its prevalence within and among countries and ethnic/heritage groups, and enhance comparison across studies. The need for consistency of methodology and for detailed disaggregated data could be addressed through the creation of repositories of deidentified data, and consortia (including representatives from research study groups, academic centers, medical societies, health authorities, and others) that would strategize the harmonization and/or standardization of methodology for data collection and analysis.

The identification of Hispanics/Latinos and Latin Americans in health-related research is inconsistent. Although the concept of a Hispanic/Latino monolith is slowly changing, the continued use of umbrella terms like “Hispanic” or “Latino” limits the ability of public health and clinical researchers to evaluate and address type 2 DM risk factors that may impact or manifest differently across Hispanic/Latino heritage groups; and this in turns delays the development of effective preventive strategies.

The etiology of type 2 DM is complex, and the research studies discussed in this review demonstrated areas in which further research could be developed. In this regard, it is not completely understood whether the oxidative stress, inflammatory and endothelial changes described in placental and umbilical cord tissue of pregnancies in which the mother had obesity or GDM could be prevented or reverted during pregnancy, and the impact that such reduction would have on the child. At the same time, although GDM has been associated with increased risk for maternal type 2 DM, it is not clear whether GDM increases the mother’s risk for CVD. Determining GDM risk (i.e., a predictive profile) for Hispanic/Latino women might identify those at risk early in pregnancy or before conception, so preventive interventions are tailored and initiated earlier.

Although obesity is associated with increased risk for type 2 DM, the distribution of adipose tissue (i.e., steatosis) has been associated to specific metabolic (421, 422) and cardiac structural abnormalities (423, 424). The study of other type 2 DM risk factors previously mentioned (e.g., sleep-disordered breathing, gut microbiome, environmental exposures) could uncover new mechanisms of disease, which can be turned into additional opportunities for interventions in Hispanic/Latino populations.

As the field of genetics/epigenetics/genomics continues evolving, additional insights on the gene-environment interaction (e.g., chronic stress, environmental endocrine disruptors, other environmental exposures) and the differences in susceptibility to type 2 DM experienced by different Hispanic/Latino heritage groups in the US and Latin America might be uncovered. Thus, the inclusion of Hispanics/Latinos in these research studies needs to improve.

The clinical assessment of Hispanics/Latinos at risk of developing type 2 DM needs to be comprehensive. The medical history needs to account underlying medical, nutritional, physical fitness, emotional, behavioral, cultural, and social factors. A detailed family history will uncover heredity patterns that could be relevant in the decision-making regarding therapy or genetic testing. The individuals’ family dynamics and social network may also uncover dietary habits and values (425–427) that could impact the disease risk, the need for pharmacological therapy, and the effectiveness of the intervention. Socioeconomic factors will influence both type 2 DM risk and the success of any prevention strategy (368–371, 427–431). Access, availability and quality of food; social and family responsibilities, social network and support; stressors; of the built environment, neighborhood safety, transportation, and access to recreational activities; availability and access to health services, health insurance type, and coverage; access to and utilization of medications and medical services; formal education attained and health literacy; and occupation(s), work shift, and occupational safety are some of the factors that will influence the success of the intervention. Incorporating these factors into the clinical assessment could uncover obstacles requiring alternative or additional interventions.

As previously discussed, Hispanics/Latinos at risk of type 2 DM often also have other cardiometabolic factors that increase the risk for developing type 2 DM or CVD (95, 146, 148, 432, 433). While lifestyle intervention would ideally be recommended to every patient at risk, some patients would also need pharmacological therapy to control other cardiometabolic factors (66, 434). Prevention of use of tobacco or its cessation should also be addressed, when applicable.

The difference in prevalence of diabetes across Hispanic/Latino heritage groups presents an opportunity to study potential genetic, biological and environmental interactions that lead to different manifestations of the disease. Deep metabolic phenotyping, (i.e., assessment of glucose homeostasis, insulin sensitivity, insulin secretion, α-cell and β-cell function, adipose tissue distribution) along with a comprehensive clinical assessment (including age, sex, clinical presentation of the disease, detailed medical history, etc.) may uncover important differences or similarities in type 2 DM phenotypes among Hispanic/Latino heritage groups, and biological or health determinants that could influence type 2 DM risk. Given the diversity of the Hispanic/Latino population, the feasibility of such enterprise could be addressed through collaborations like those mentioned above, exploring the use of clinical databases, and creating patient/data registries, among other potential collective efforts.

Type 2 diabetes mellitus preventive strategies have mostly focused on weight reduction, and have produced promising results. However, given the increasing prevalence of obesity in the Hispanic/Latino populations, type 2 DM diabetes and obesity preventive efforts may require even more intensive approaches, perhaps through risk stratification. Preventive efforts focusing on those at higher risk due to age, health status, medications, sedentary lifestyle, family history of type 2 DM or GDM might be different than the strategies for those who have lower susceptibility. The timing of prevention should also be considered. Earlier onset of obesity has been associated with higher risk of developing type 2 DM (173–175), for example. Also, risk stratification should be carefully determined and considering all factors mentioned above. For instance, the DPP reported that over a 10-year follow-up period from randomization, the initial differences in incident diabetes among treatment arms, including the lifestyle intervention, began to narrow (435). Through a retrospective analysis, the investigators examined participants’ susceptibility to type 2 DM, including genetic risk scores (GRSs) (435, 436). The investigators concluded that although clinical and genetic susceptibility could influence risk to develop type 2 DM, GRS alone did not determine the success of the lifestyle intervention; at the highest quartile of GRS, intensive lifestyle intervention was effective at reducing risk (435, 436).

Few behaviors are as closely tied to culture as diet and self-care. What, when, and how much we eat, as well as our notions of health and disease, including whose advice we seek, are firmly grounded in the cultural context which shapes us. It may influence beliefs about what causes diabetes, whether to engage in type 2 DM risk-reduction activities, seek advice regarding the disease, and decide which treatment options are followed. Despite their impact on health and disease management, cultural considerations are often an afterthought in the development and implementation of preventive or treatment interventions targeting type 2 DM in Hispanic/Latino populations. A recent review found only 12 published studies on behavioral interventions aimed at reduction of type 2 DM and specifically designed for Hispanic/Latino adults (380). All interventions were offered in Spanish and took place in community settings, and nine included materials for individuals of low literacy. However, only two included Hispanic/Latino foods or recipes, community input in the intervention content, and addressed cultural beliefs regarding diabetes. Thus, it appears that for most studies, the process of infusing culture into interventions remains at the surface structures (language and setting in which intervention is delivered), without fully incorporating cultural values and elements into intervention messages, materials, design, and implementation (437). Given the evidence that culturally coherent behavioral interventions for diabetes prevention/management among Hispanic/Latino groups are more effective than usual care in improving HbA1c, weight/body mass index, lipid, blood pressure, dietary intake, PA, and diabetes knowledge (438), there is a strong case for ensuring the presence of cultural-competency components in every diabetes-related healthcare service intervention. Cultural competence must be at the core of clinical care and the design and implementation of research studies and large-scale interventions.

Lifestyle intervention studies to prevent type 2 DM in Hispanics/Latinos have mostly been focused on short-term goals of weight reduction, and increasing PA. While they have demonstrated modest success at meeting these goals, follow-up periods have been insufficiently long to demonstrate prevention or delay of type 2 DM. Initiating lifestyle interventions before pregnancy (to prevent GDM, for example), and improving representation of women of various Hispanic/Latino heritage groups, should be seriously considered. Studies focused on children and adolescents could explore the impact of factors, such as maternal health, prenatal care, and child’s development on preventive interventions. Advances in the field will require funding opportunities that allow for longer follow-up periods, permit the examination of interventions and outcomes for different Hispanic/Latino populations, and allow for comparisons of various elements of cultural adaptation. On the other hand, integrating research interventions within established clinical settings or health systems could be resource-efficient way to implement research studies on type 2 DM prevention, including the follow-up of research study participants.

Quality of life and savings in disability-adjusted life years resulting from delaying the onset of type 2 DM should be quantified and qualified, especially among high-risk populations like Hispanics/Latinos. Also, the evaluation of type 2 DM risk prevention in multilevel community studies to prevent childhood obesity (331, 332), coupled with mass media campaigns to increase awareness of type 2 DM prevention (336, 439), and other risk factors for diabetes, is warranted.

The success of large culturally tailored interventions, such as those modeled after the DPP, underscore the promise that such interventions might result in improved quality of life, higher productivity, and reduced morbidity and mortality for Hispanic/Latino populations. However, it is critical to remain aware that the vast heterogeneity in the Hispanic/Latino population requires clinicians and researchers alike to avoid relying on vague and largely unexamined concepts, such as *familism* and *marianism* without first verifying their relevance in the population of interest. Often, the uncritical acceptance of these values as omnipresent in individuals of Hispanic/Latino origin results in the automatic assumptions that, for example, interventions must be family based. Upon closer examination, studies have found that the Hispanic family unit is much more complex, and less unconditionally supportive, than it is often assumed to be, and in many cases, some Hispanic/Latino women prefer women-only group interventions without the inhibiting presence of family members, children, or spouses (440, 441).

In both clinical and research programs, engagement of stakeholders, not only including the patient population and the healthcare provider team, but also administrative clinic and research staff, is critical for the success of health-promotion interventions. A bidirectional communication style, from clinicians/investigators to patients and community, is necessary to ensure the cultural coherence, accessibility, and sustainability of a program. Individuals who are thoroughly competent in the cultural, socioeconomic, and even geographic context of the target population can navigate cultural nuances of these heterogeneous populations and provide invaluable guidance in the design and implementation of both large- and small-scale preventive and treatment efforts.

Upon designing a research project or implementing a clinical program to prevent type 2 DM in Hispanics/Latinos in the US and in Latin America, other challenges need to be acknowledged. Insufficient institutional (government, academic, etc.) interest or commitment, lack of public awareness on the importance of prevention, insufficient financial and human resources, and political issues are some of the challenges faced across the continent (442–444). Whereas out of the scope of this review, proposed models of an international collaborative initiative (442) and of an implementation cycle and a translatability scale for identifying and implementing effective solutions to address the rising burden of chronic diseases in low-middle income countries, specifically CVD (444), might set the stage for future type 2 DM prevention initiatives in the continent.

The progressive and sustained increase in type 2 DM among Hispanics/Latinos is a clear indicator that preventive efforts need to be seriously and urgently reframed. This will require a better understanding of the extent and etiology of the disease, creating solutions through multidisciplinary approaches, and a fundamentally radical transformation in our priorities as individuals and as communities.

## Author Contributions

MLAS conceptualized the manuscript. MLAS, UCR, FJP, JM, NML and CMP researched the literature, wrote different sections of the manuscript, reviewed the manuscript in its entirety, and approved its final version. MLAS and NML did the final review and editing of the manuscript.

## Disclaimer

The views expressed in this manuscript are those of the authors and do not necessarily represent the views of the National Heart, Lung, and Blood Institute; the National Institutes of Health; or the US Department of Health and Human Services.

## Conflict of Interest Statement

The authors declare that the research was conducted in the absence of any commercial or financial relationships that could be construed as a potential conflict of interest.

## Appendix

**Table:**

**Abbreviations**	
AA(s)	African American(s)
ACE inhibitor	angiotensin-converting enzyme inhibitor
AD	Alzheimer’s disease
ARB	angiotensin II receptor blocker
ADA	American Diabetes Association
AHEI	alternate health eating index
BCAAs	branched-chain amino acids
BMI	body mass index
BRFSS	Behavioral Risk Factor Surveillance Survey
BW	birth weight
CARMELA	cardiovascular risk factor multiple evaluation in Latin America
CESCAS I	*Centro de Excelencia en Salud Cardiovascular para el Cono Sur I*
CRONICAS	*Centro de Excelencia de Enfermedades Crónicas*
CRP	C reactive protein
CV	cardiovascular
CVD	cardiovascular disease
DASH	dietary approaches to stop hypertension
DEBI	diet exercise and breastfeeding intervention
DM	diabetes mellitus
DNA	deoxyribonucleic acid
DPP	Diabetes Prevention Program
ELSA-Brasil	*Estudo Longitudinal de Saúde do Adulto*
FFA	free fatty acids
FBG	fasting blood glucose
FPG	fasting plasma glucose
FSIGT	frequently sampled intravenous glucose tolerance test
FTO	alpha-ketoglutarate dependent dioxygenase
GDM	gestational diabetes mellitus
GEM	gestational diabetes’ effects on moms
GRS	genetic risk score
GUARDIAN	genetics underlying diabetes in Hispanics
GWAS	genome wide association studies
HbA1c	hemoglobin A1c
HCHS/SOL	Hispanic Community Health Study/Study of Latinos
HDL-C	high-density lipoprotein cholesterol
Hispanic HANES	Hispanic Health and Nutrition Examination Survey
HOMA-IR	homeostasis model assessment for insulin resistance
IDF	International Diabetes Federation
IGT	impaired glucose tolerance
IL-6	interleukin 6
KPD	ketosis prone diabetes
LASO	Latin American Consortium of Studies in Obesity
LDL-C	low-density lipoprotein cholesterol
MCP-1	macrophage chemoattractant protein 1
MESA	Multi-Ethnic Study of Atherosclerosis
NCEP	National Cholesterol Education Program
NHANES	National Health and Nutrition Examination Survey
NHIS	National Health Interview Survey
NHB(s)	non-Hispanic black(s)
NHW(s)	non-Hispanic white(s)
OGTT	oral glucose tolerance test
OMB	office of management and budget
OSA	obstructive sleep apnea
PA	physical activity
PAHO	Pan American Health Organization
PPAR	peroxisome proliferator-activated receptor
RCT(s)	randomized clinical trial(s)
REACH	racial and ethnic approaches to community health
SAHS	San Antonio Heart Study
SNPs	single-nucleotide polymorphisms
Type 1 DM	Type 1 diabetes mellitus
Type 2 DM	Type 2 diabetes mellitus
TGs	triglycerides
TCF7L2	transcription factor 7-like 2
TNF-α	tumor necrosis factor alpha
US	United States
WHO	World Health Organization
